# A comprehensive aerobiological study of the airborne pollen in the Irish environment

**DOI:** 10.1007/s10453-022-09751-w

**Published:** 2022-07-28

**Authors:** Emma Markey, Jerry Hourihane Clancy, Moisés Martínez-Bracero, Jose María Maya-Manzano, Matt Smith, Carsten Skjøth, Paul Dowding, Roland Sarda-Estève, Dominique Baisnée, Aoife Donnelly, Eoin McGillicuddy, Gavin Sewell, David J. O’Connor

**Affiliations:** 1 School of Chemical and Pharmaceutical Sciences, Technological University Dublin, Dublin, Ireland; 2 Center of Allergy and Environment (ZAUM), Member of the German Center for Lung Research (DZL), Technische Universität München/Helmholtz Center Munich, Munich, Germany; 3 School of Science and the Environment, University of Worcester, Worcester, UK; 4 Trinity Centre for the Environment, Trinity College Dublin, Dublin, Ireland; 5 Laboratoire des Sciences du Climat et de l’Environnement (LSCE), CNRS-CEA-UVSQ, Gif-sur-Yvette, France; 6 School of Food Science and Environmental Health, Technological University Dublin, Dublin, Ireland; 7 School of Chemical Sciences, Dublin City University, Dublin, Ireland

**Keywords:** Aerobiology, Allergens, Pollen monitoring, Pollen calendar, Meteorology, Ireland

## Abstract

Respiratory allergies triggered by pollen allergens represent a significant health concern to the Irish public. Up to now, Ireland has largely refrained from participating in long-term aerobiological studies. Recently, pollen monitoring has commenced in several sampling locations around Ireland. The first results of the pollen monitoring campaigns for Dublin (urban) and Carlow (rural) concerning the period 2017–2019 and 2018–2019, respectively, are presented herein. Additional unpublished pollen data from 1978–1980 and, 2010–2011 were also incorporated in creating the first pollen calendar for Dublin. During the monitoring period over 60 pollen types were identified with an average Annual Pollen Integral (APIn) of 32,217 Pollen × day/m^3^ for Dublin and 78,411 Pollen × day/m^3^ for Carlow. The most prevalent pollen types in Dublin were: Poaceae (32%), Urticaceae (29%), Cupressaceae/Taxaceae (11%), *Betula* (10%), *Quercus* (4%), *Pinus* (3%), *Fraxinus* (2%), *Alnus* (2%) and *Platanus* (1%). The predominant pollen types in Carlow were identified as Poaceae (70%), Urticaceae (12%)*, Betula* (10%), *Quercus (2%), Fraxinus* (1%) and *Pinus* (1%). These prevalent pollen types increased in annual pollen concentration in both locations from 2018 to 2019 except for *Fraxinus.* Although higher pollen concentrations were observed for the Carlow (rural) site a greater variety of pollen types were identified for the Dublin (urban) site. The general annual trend in the pollen season began with the release of tree pollen in early spring, followed by the release of grass and herbaceous pollen which dominated the summer months with the annual pollen season coming to an end in October. This behaviour was illustrated for 21 different pollen types in the Dublin pollen calendar. The correlation between ambient pollen concentration and meteorological parameters was also examined and differed greatly depending on the location and study year. A striking feature was a substantial fraction of the recorded pollen sampled in Dublin did not correlate with the prevailing wind directions. However, using non-parametric wind regression, specific source regions could be determined such as *Alnus* originating from the Southeast, *Betula* originating from the East and Poaceae originating from the Southwest.

**Supplementary Information:**

The online version contains supplementary material available at 10.1007/s10453-022-09751-w.

## Introduction

Exposure to pollen is a major trigger for people with allergic disorders such as asthma (D’Amato et al., 2020; D’Amato et al., 2007b; Taylor et al., 2002). Ireland has one of the highest hospital discharge and death rates associated with asthma in Western Europe and the 4th highest incidence rate worldwide with 60–80% of Irish asthmatics also suffering from allergic rhinitis (Asthma Society of Ireland, 2020). With trends in pollen allergy prevalence expected to drastically increase in coming decades, further accelerated by climate change (Lake et al., 2017), it is becoming increasingly important to develop and maintain an active pollen monitoring network in Ireland. Airborne pollen can also directly influence the climate and hydrological cycle by forming ice nuclei which affect cloud formation, thus altering radiative forcing (Després et al., 2012; Diehl et al., 2001; Pummer et al., 2012). Despite these significant health and climatic implications, Ireland does not currently possess a fully operational pollen monitoring network. Existing regional differences in airborne pollen concentrations between European countries have highlighted the need for further pollen monitoring expansion (Leru et al., 2018; Sikoparija et al., 2017). Therefore, the pollen forecasts available to the Irish public, thus far, may not be fully representative of the airborne pollen concentrations present in the environment as they are estimated by monitoring networks in the UK.

Other European countries have been routinely monitoring pollen for decades, leading to the formation of the European Aeroallergen Network (EAN) in 1986 (Nilsson, 1988). Since the initial formation of the EAN with 251 sampling sites, the extent of pollen monitoring in Europe has greatly expanded to over 525 sampling sites (Buters et al., 2018). Although Ireland was one of the original 21 countries to join the EAN, further progress was interrupted due to the initial monitoring efforts being adjourned in the early 1980s. Thus, the prevalent species and seasonality of the different pollen types in the Irish environment are not yet known due to failure to conduct preliminary surveys similar to those conducted in mainland Europe and the UK during the 1980–1990’s (Belmonte & Roure, 1991; Emberlin et al., 1990; Giner et al., 1999; Spieksma, 1983, 1986; Spieksma et al., 1985).

Two limited aerobiological studies previously carried out in Ireland focused on investigating the influence of weather conditions on selected pollen and fungal spore types (McDonald, 1980; McDonald & O’Driscoll, 1980) but provided little detail on the various pollen types recorded. In the last decade, field campaigns have been conducted in various locations around Ireland using the Wideband Integrated Bioaerosol Sensor (WIBS) to monitor primary biological aerosol particles (PBAP) such as fungal spores (Healy et al., 2012, 2014; O’Connor et al., 2013, 2015a, 2015b) and pollen (Healy et al., 2012; O’Connor et al., 2014a, 2014b), but the campaigns were relatively short and provided little information on the seasonal concentrations and trends of PBAP in Ireland.

Propelled by the prevalence of allergic and respiratory diseases in Ireland, pollen monitoring has recently recommenced in Ireland under the auspices of the pollen Monitoring and Modelling (POMMEL) project. There remains a considerable disparity between Ireland and other European aerobiological monitoring networks (Buters et al., 2018; Maya-Manzano et al., 2021). Therefore, this study represents one of the first compiled analyses of the existing airborne pollen types in two different locations in the Irish environment. More recently, there have been efforts to further extend such traditional monitoring studied within Ireland. These studies have examined the spatiotemporal variations in the distribution of birch trees and *Betula* pollen (Maya-Manzano et al., 2021) and the use of historical data for fungal spore monitoring (Martínez-Bracero et al., 2022).

The primary aim of this study is to analyse pollen data collected at two separate sampling sites in Ireland to compile a detailed aerobiological survey of the prevalent pollen types and their seasonality. A comparison between the urban sampling site situated in Dublin City centre and the more rural locale in Carlow will also be made to address any deviations in pollen type and concentration. Continuing from preliminary studies carried out in the 1970s, a more comprehensive investigation between pollen count and meteorological data will also be conducted. Finally, an initial pollen calendar for Dublin will be presented using the pollen data collected during 2017–2019 and the unpublished data from 1978–1980 and 2010–2011. This study represents an imperative starting point in re-establishing an Irish pollen monitoring network.

## Material and methods

### Sampling locations

**Fig. 1:**
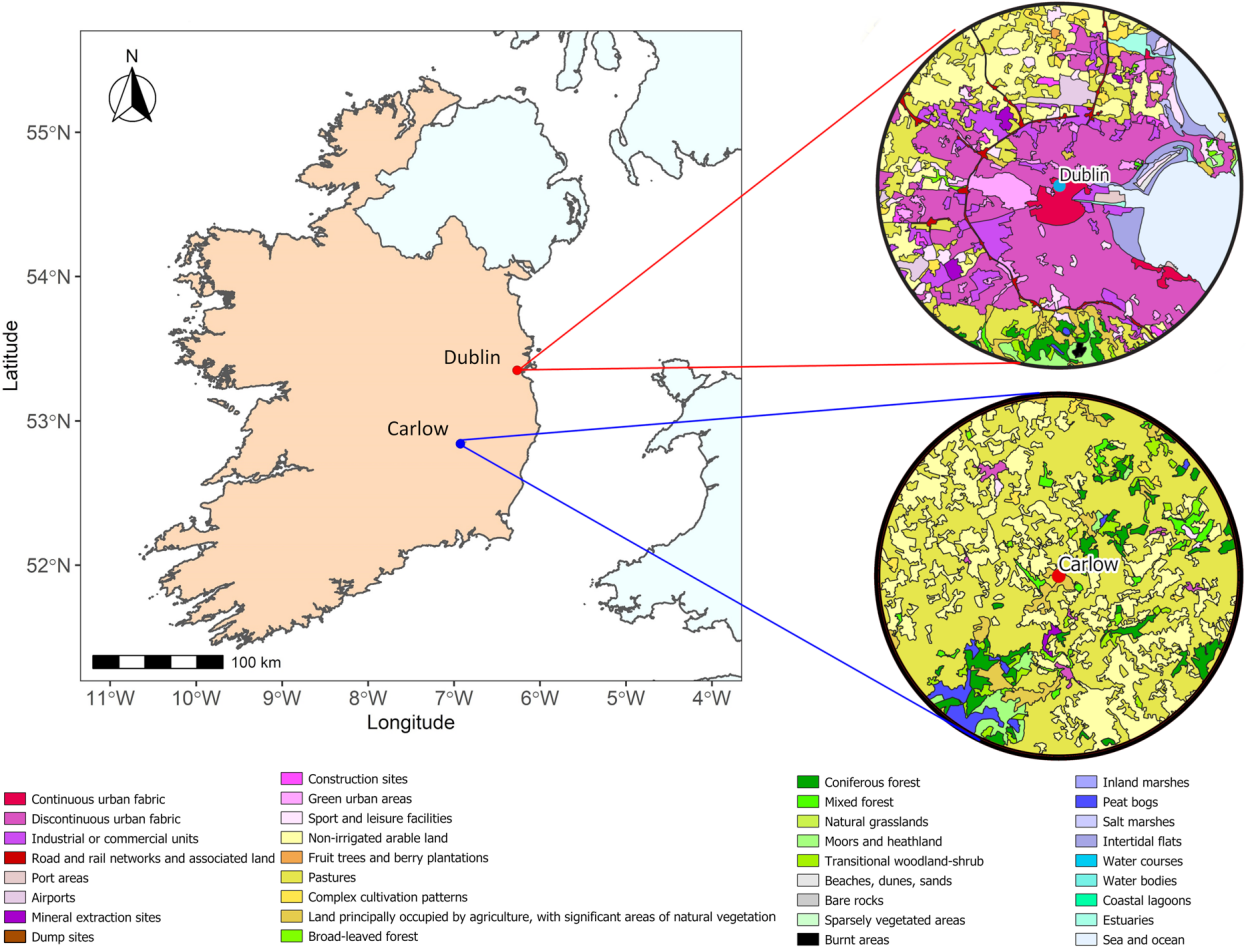
Map of sampling locations in Ireland (Dublin—Red, Carlow–Blue) with a magnified site map detailing the and cover of both sampling sites, covering a distance of 30 km

The aerobiological survey was carried out in the province of Leinster, located in the east of Ireland, using data collected from two sites (Fig. 1), one rural (Carlow), and one urban (Dublin). Further detail regarding the land cover surrounding both sites is also provided in Fig. 1. The surveyed region possesses a cool temperate oceanic climate, with mean annual temperatures between 10 and 10.5 °C. Rainfall in this region is the lowest in the country, with an average annual rainfall of 750 mm to 1000 mm per year, compared to a national average of over 1200 mm annually. The Dublin site (53°20′12.1ʺ N, 6°16′04.0ʺ) is located on the roof of the Technical University Dublin (20 m high), in the centre of a coastal metropolitan area covering approximately 318km^2^ (CSO, 2012) with a population density of 4811/km^2^ (CSO, 2016). The Carlow site (52°43′23.6ʺ N, 6°39′36.0ʺ W) has a population density of 63/km^2^ (CSO, 2016), and is a rural, agricultural, inland area, with its pollen trap located atop a pedestal (2 m high) on a farm.

### Pollen monitoring and analysis

Pollen data was collected using Hirst-Lanzoni 7-day pollen sampler (Hirst, 1952). The samplers drew in air at a rate of 10L/min in which the pollen was impacted upon a silicone-coated tape (Lanzoni). Each seven-day tape was cut and mounted as daily pollen slides and counted manually using an optical microscope at 400 × magnification. Four horizontal transects per slide were counted (equating to 12–13% of total surface area) according to the standardized methods for airborne pollen monitoring counting and analysis suggested by the Spanish Aerobiology Network (REA) and the minimum requirements of the European Aerobiological Network (Galán et al., 2007, 2014; Tormo-Molina et al., 1996).

The raw pollen count was converted into daily concentrations (pollen grains/m^3^) and the annual pollen integral (APIn) was calculated for each pollen type by multiplying the average daily concentration of the annual sampling period by the season duration, using the recommended aerobiological method (Galán et al., 2017). The term “pollen type” refers to pollen grains sharing the same morphological characteristics observed under microscopic analysis. This includes pollen grains belonging to different taxonomical categories—either a specific genus or family of plant, with all respective species included within. The major pollen types were defined as the pollen types that possessed an annual pollen integral of greater than or equal to 100 Pollen * day/m^3^ (Galán et al., 2017). The Main Pollen Season (MPS) for each pollen type was calculated as the start and end dates where the annual pollen concentration sum reached 5% and 95% of the total for the year (Cristofori et al., 2010; Nilsson & Persson, 1981). Several methods can be used to calculate the MPS (Jato et al., 2006). The 90% method of determining the MPS was selected due to its capability to suit plants/trees that pollinate early in the year (Kasprzyk, 2003; Nilsson & Persson, 1981), as well as allowing the MPS to be determined while excluding low concentrations that could be transported from other regions (Kasprzyk et al., 2004) such as the UK.

Pollen monitoring was conducted from 22nd of May 2017 continuously until the 30th of September 2019 in Dublin. Pollen monitoring in Carlow took place from the 18th of April until the 10th of December 2018 and recommenced on the 1st of February until the 30th of September 2019.

### Meteorological data

Meteorological data were obtained from the Met Éireann website (The Irish National Meteorological Service 2020). Weather stations in both Carlow in Oak Park (52°51′40.0ʺ N, 6°54′55.0ʺ W, 22.5 km from sampling site, 62 masl), and Dublin in Dublin Airport (53°25′40.0ʺ N 6°14′27.0ʺ W, 11 km from the sampling site, 74 masl) provided the following daily datasets of major meteorological parameters. All parameters represent daily means unless otherwise stated. For Carlow, the available parameters were: Mean Temperature [ºC] (*T*_mean_), Maximum Temperature [°C] (*T*_max_) and Minimum Temperature [°C] (*T*_min_), Average Mean Temperature [°C] over the previous 10 days (*T*_mean_10_), Grass Minimum temperature [°C], 2 cm above ground (Gmin), Mean 10 cm soil temperature [°C] (Soil), Precipitation amount [mm] (Rain), Average Precipitation amount [mm] over the previous 10 days (Rain_10), Mean CBL Pressure [hpa] (Cbl), Mean Wind Speed [kt] (Wind_s) and Wind direction at max 10 min mean [deg] (Wind_d), global radiation [J/cm^2^] (G_rad), Potential Evapotranspiration [mm] (Pe), Evaporation [mm] (Evap) and relative humidity [%] (Rh). The same parameters plus the sunshine duration [hours] (Sun) were used for Dublin.

### Land cover data

Land cover maps were generated for each sampling location within a 30 km radius of the sampling site. This range is considered to reflect the overall pollen distribution and types recorded when using a volumetric trap (Skjøth et al., 2010). The source maps were then generated using CORINE Land cover data (Basu, 2021; Büttner et al., 2004).

### Statistical analysis

The normal distribution of the data was tested using the Lilliefors test, one variation of the Kolmogorov–Smirnov test. This test is typically recommended for larger sampler sizes as an alternative to the commonly used Shapiro-Wilks test, while both tests are commonly used in aerobiology (Galán et al., 2014; Grinn-Gofroń et al., 2015; Orlandi et al., 2014; Picornell et al., 2020). These tests showed that the daily data did not follow a normal distribution, even after the logarithmic transformation. A Spearman correlation test was then used to calculate the degree and the significance between selected variables. The statistical analysis included only days within the MPS and was performed by use of the packages *nortest* (Gross & Ligges, 2015) and *corrplot* (Wei et al., 2021) in the software R (R Core Team, 2017).

### Geographical location of airborne pollen

A source receptor approach was used to establish the geographical origin of airborne pollen observed in Dublin. The approach uses ambient airborne pollen concentrations and observed wind data using the software package ZeFir-v3.7 (Petit et al., 2017). A variant of the two-dimension Non-parametric Wind Regression (NWR) known as Sustained Wind Incidence Method (SWIM) was used, originally developed by Henry et al. (2009) and later adapted by Olson et al. (2012), known as the Sustained Wind Incidence Method (SWIM). This method has been successfully applied to determine the geographical origins of pollen, fungal spores and bacteria (Sarda-Estève et al., 2018, 2019, 2020). The approach is similar to wind air pollution roses but takes the standard deviation of the wind speed and the wind direction into account.

Equation (1) describes the calculation of SWIM (Si):

1 $$S_{i} = \frac{{C_{i} \cdot \Upsilon_{i} }}{{\max \left( {C_{i} \cdot \Upsilon_{i} } \right)}} \cdot \frac{{\overline{\delta }}}{{\delta_{i} }}$$

where *C*_*i*_, *γ*_*i*_ and *δ*_*i*_ represent wind speed, wind direction, and wind direction standard deviation, respectively. This allows for the analysis of daily pollen concentration values along with the highly variable wind speed and wind directions. Wind direction standard deviation was estimated by the 1-pass Yamartino equations (Yamartino, 1984), which is part of the ZeFir package.

### Pollen calendar

Data from unpublished pollen monitoring campaigns from 1978–1980, 2010–2011 and from the POMMEL monitoring campaign (2017–2019) were combined to construct the first pollen Calendar for Dublin. The mean daily pollen values for all years were calculated for 21 reoccurring pollen types that were present for all sampling years. Daily values for each month were further divided into 5 sections per month, containing 6 days each. The mean value for each section was then calculated. This was repeated for each pollen type. Early, late and main flowering periods were calculated using the method suggested by Werchan et al. (2018). The main flowering period was defined as the 10–90% interval, beginning once the pollen interval reached 10% of the APIn and ending once 90% was reached. Early and late flowering periods, outside the main flowering period, were determined in a similar manner. The early flowering period defined the period within the interval where the pollen integral exceeded 0.5% of the APIn but is less than 10%. Similarly, the late flowering period corresponded to the period within the interval where the pollen integral exceeded 90% of the APIn but less than 99.5%. Finally, possible occurrence times were determined as any time outside of the 0.5–99.5% range where pollen was observed. The pollen calendar was then constructed and coloured according to the level of allergenicity posed by each pollen type and shaded according to possible, early/late, and main flowering periods.

## Results

### Pollen data

During the continuous sampling period of 2017–2019 (Dublin) and 2018–2019 (Carlow), 61 different pollen types were identified in the Irish environment. 31 were classified as herbaceous/grass in nature and 30 as arboreal in nature. The main pollen types varied slightly in both locations. The predominant pollen types in Dublin were identified as Poaceae (32%), Urticaceae *(*29%)*,* Cupressaceae/Taxaceae (11%)*, Betula* (10%)*, Quercus* (4%)*, Pinus* (3%), *Fraxinus* (2%)*, Alnus* (2%) and *Platanus* (1%) which represented 93% of the overall pollen during the sampling period. Similarly, the most abundant pollen types in Carlow were identified as Poaceae (70%), Urticaceae (12%)*, Betula* (5%)*, Quercus* (2%)*, Fraxinus* (1%) and *Pinus* (1%) which represented 91% of the pollen encountered during the sampling period.

**Fig. 2:**
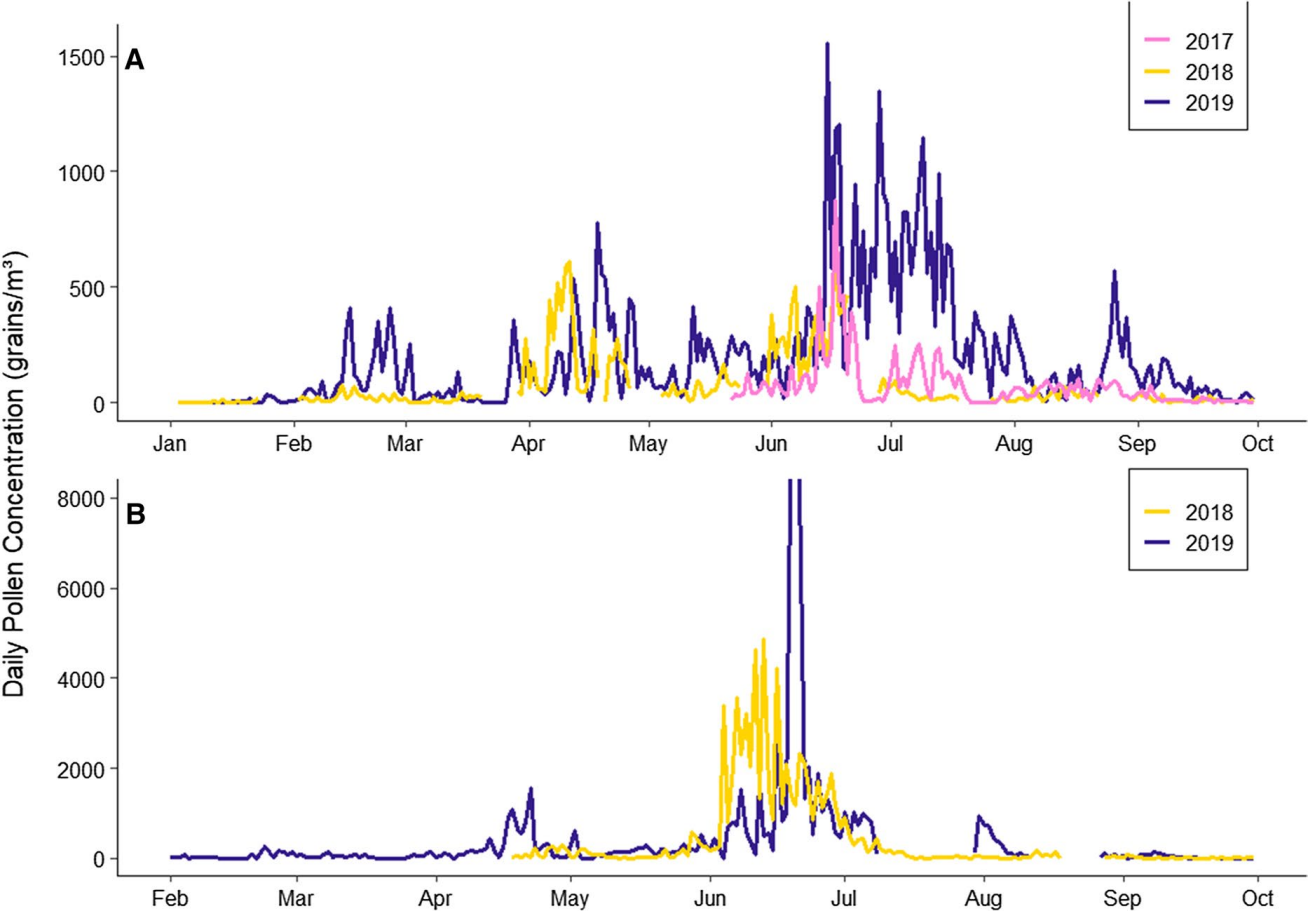
Daily total pollen concentration in **A** Dublin (2017–2019) and **B** Carlow (2018–2019)

The general trends in the pollen season began at the end of January/start of February and lasted until the end of September (Fig. 2). The annual pollen season in Ireland commenced with the release of arboreal pollen, firstly with *Corylus*, followed by *Alnus,* Cupressaceae/Taxaceae and *Betula*. Other tree pollen types were also present during later Spring months such as *Pinus, Salix, Platanus, Populus, Fraxinus* and *Quercus* as the main pollen season of *Corylus* and *Betula* came to an end around May/June. Throughout the monitoring campaigns, the end of spring saw the commencement of Poaceae pollination, generally accompanied by other herbaceous pollen types such as *Rumex, Plantago* and Urticaceae and several arboreal pollens such as *Tilia* and *Castanea *and continuing concentrations of Cupressaceae/Taxaceae and *Pinus.* This general trend, observed in both locations, can be seen through the sequence and seasonal duration of the main pollen types (Tables 1, 2).

**Table 1:** Major Pollen types and Main Pollen Season Parameters for Dublin 2018–2019

Major Pollen	Start Date	End Date	Duration of Season (Days)	Max Daily Concentration (grains/m^3^)	Day of Max Concentration	APIn (Pollen * day/m^3^)	% of Total Pollen
Dublin 2018							
*Alnus*	03/02/2018	31/03/2018	56	17	12/02/2018	244	1.5%
*Betula*	06/04/2018	25/04/2018	19	346	11/04/2018	2623	16.2%
*Corylus*	14/01/2018	02/04/2018	78	17	16/02/2018	109	0.7%
Cupressaceae/Taxaceae	14/02/2018	09/07/2018	145	208	10/04/2018	3063	18.9%
*Fraxinus*	31/03/2018	23/04/2018	23	218	11/04/2018	1549	9.5%
*Pinus*	06/04/2018	08/06/2018	63	43	20/05/2018	304	1.9%
Poaceae	20/05/2018	05/07/2018	46	410	17/06/2018	3588	22.1%
*Quercus*	22/04/2018	29/07/2018	98	18	10/05/2018	331	2.0%
Urticaceae	01/06/2018	03/09/2018	94	257	19/06/2018	3535	21.8%
Dublin 2019							
*Alnus*	27/01/2019	02/03/2019	34	168	15/02/2018	1495	2.9%
*Betula*	28/03/2019	11/05/2019	44	553	18/04/2018	4905	9.4%
*Castanea*	29/06/2019	04/08/2019	36	179	16/07/2019	413	0.8%
*Corylus*	21/01/2019	02/03/2019	20	12	15/02/2019 22/02/2019	157	0.3%
Cupressaceae/Taxaceae	14/02/2019	28/06/2019	134	243	25/02/2019	5108	9.8%
Ericaceae	18/06/2019	10/09/2019	84	29	28/08/2019	118	0.2%
*Fagus*	19/04/2019	14/05/2019	25	62	26/04/2019	187	0.4%
*Fraxinus*	19/03/2019	17/05/2019	59	14	18/04/2019	168	0.3%
*Pinus*	05/05/2019	29/06/2019	55	125	22/05/2019	1621	3.1%
*Plantago*	15/05/2019	26/08/2019	105	18	21/06/2019	372	0.7%
*Platanus*	04/04/2019	07/05/2019	33	89	09/04/2019	502	1.0%
Poaceae	07/06/2019	01/08/2019	55	1057	15/06/2019	17350	33.3%
*Quercus*	22/04/2019	05/07/2019	73	152	11/06/2019	2696	5.2%
*Rumex*	22/05/2019	22/07/2019	60	25	07/07/2019	345	0.7%
*Salix*	26/02/2019	27/04/2019	60	16	31/03/2019	225	0.5%
*Ulmus*	15/02/2019	31/03/2019	44	28	01/03/2019	133	0.3%
Urticaceae	10/06/2019	08/09/2019	90	534	26/08/2019	14778	28.4%

**Table 2:** Major Pollen types and Main Pollen Season Parameters for Carlow 2018–2019

Major Pollen	Start Date	End Date	Duration of Season (Days)	Max Daily Concentration (grains/m^3^)	Day of Max Concentration	APIn (Pollen * day/m^3^)	% of Total Pollen
Carlow 2018							
*Betula*	23/04/2018	05/06/2018	43	125	04/05/2018	800	1.08%
*Castanea*	09/06/2018	10/07/2018	31	35	01/07/2018	195	0.26%
Cupressaceae/Taxaceae	21/04/2018	25/09/2018	157	20	27/04/2018	250	0.34%
*Fraxinus*	21/04/2018	07/05/2018	16	240	29/04/2018	1585	2.13%
*Pinus*	22/05/2018	20/06/2018	29	174	28/05/2018	761	1.03%
*Plantago*	30/05/2018	31/07/2018	62	33	10/06/2018	490	0.67%
Poaceae	04/06/2018	04/07/2018	30	4659	13/06/2018	52776	71.41%
*Quercus*	22/05/2018	10/07/2018	49	74	28/05/2018	788	1.07%
*Rumex*	30/05/2018	27/06/2018	28	43	11/06/2018	558	0.75%
Urticaceae	04/06/2018	17/08/2018	74	429	28/06/2018	7538.7	10.33%
Carlow 2019							
*Alnus*	07/02/2019	16/03/2019	51	222	22/02/2019	1392	1.65%
*Betula*	08/04/2019	01/05/2019	27	1385	22/04/2019	7309	8.67%
*Carpinus*	19/04/2019	08/05/2019	19	47	26/04/2019	269	0.32%
*Corylus*	04/02/2019	27/03/2019	53	41	04/02/2019	228	0.27%
Cupressaceae/Taxaceae	23/02/2019	31/05/2019	66	101	08/03/2019	1334	1.58%
*Fraxinus*	27/03/2019	16/05/2019	50	23	02/05/2019	211	0.25%
*Pinus*	26/04/2019	02/06/2019	37	123	18/05/2019	1125	1.34%
*Plantago*	19/05/2019	30/06/2019	42	437	19/06/2019	2322	2.76%
Poaceae	08/06/2019	03/07/2019	25	19204	20/06/2019	55364	65.69%
*Quercus*	07/04/2019	02/06/2019	56	185	02/05/2019	1825	2.17%
*Rumex*	05/06/2019	29/06/2019	24	99	20/06/2019	887	1.05%
Urticaceae	03/06/2019	07/08/2019	65	1074	16/06/2019	10522	12.32%

In Dublin 2018, 9 major pollen types were identified, this increased to 17 major pollen types in 2019, including the previous 9. These reoccurring 9 pollen types were: *Alnus, Betula, Corylus,* Cupressaceae/Taxaceae*, Fraxinus, Pinus,* Poaceae, *Quercus* and Urticaceae. Deviations in MPS parameters were observed for these pollen types between 2018 and 2019. In 2019, *Alnus* pollination commenced sooner, saw an increase in the maximum observed daily concentrations and increase in APIn but had a shorter seasonal duration. The MPS of *Betula* also started earlier in 2019, saw an increase in maximum daily concentration and APIn but unlike *Alnus*, had a longer seasonal duration. Conversely, *Corylus* MPS commenced one week later in 2019, had a shorter seasonal duration than in 2018 and saw a decrease in peak daily concentrations but had an increase in APIn. Cupressaceae/Taxaceae pollen season commenced on the same day in 2019 and 2018, however, 2019 saw an earlier finish with an increase in peak daily concentrations and APIn*. Quercus* illustrated the same trend as Cupressaceae/Taxaceae with the same start date for both years, a reduction in season durations and an increase in peak daily concentration and APIn. *Pinus* and Urticaceae also illustrated comparable differences from 2018 to 2019. Both MPSs commenced later in 2019, with *Pinus* commencing nearly a month later than in 2018. Both MPSs had a shorter seasonal duration in 2019 but saw an increase in daily peak concentrations and APIn. Poaceae also had a later MPS start date in 2019 of 18 days but had a longer seasonal duration of an extra 9 days with a sharp increase in daily peak airborne pollen concentration and APIn. Whereas all aforementioned pollen types increased in overall annual concentration, one pollen type in both Dublin and Carlow illustrated a sharp decrease in annual pollen concentration. The MPS of *Fraxinus* began 12 days earlier in Dublin 2019 and 25 days earlier in Carlow 2019 in comparison to the previous year. The seasonal duration in both locations saw a dramatic increase from 23 to 59 days in Dublin and from 16 to 50 days in Carlow, this extended pollen season was also accompanied by a sharp decline in daily peak concentration and APIn. The 2018 APIn of *Fraxinus* was 1549 Pollen × day/m^3^ and 1585 Pollen × day/m^3^ in Dublin and Carlow, respectively. These values declined to 168 Pollen × day/m^3^ and 211 Pollen × day/m^3^ in Dublin and Carlow 2019.

Similar trends for other major pollen types were also observed in Carlow. In 2018, 10 major pollen types were classified which increased to 12 in 2019, including 9 pollens from the previous year. *Alnus* and *Corylus* were not sampled in early 2018 in Carlow due to discrepancies at the monitoring location, however, they were present in considerable concentrations in 2019. The 9 repeating main pollens from 2018 to 2019 include *Betula,* Cupressaceae/Taxaceae, *Fraxinus, Pinus, Plantago*, Poaceae, *Quercus, Rumex* and Urticaceae. The MPS’s of *Betula* and Cupressaceae/Taxaceae both commenced earlier in 2019 than in the previous year, showed an increase in daily peak concentration and APIn but the duration of both seasons was reduced by 16 and 91 days respectively. The MPS’s of *Quercus, Plantago* and Urticaceae commenced earlier in 2019 and had shorter seasonal durations but saw an increase in peak daily concentrations and APIn. Similarly, Poaceae and *Rumex* both commenced pollinations later in 2019 than in 2018 and had a shorter seasonal duration but again saw an increase in peak daily concentration and APIn. Lastly, the MPS of *Pinus* began almost a month earlier in 2019 than in 2018 and saw an increase in seasonal duration from 29 to 37 days and an increase in APIn but saw a reduction in peak daily concentrations from 174 Pollen × day/m^3^ to 123 Pollen × day/m^3^.

The seasonal variation of peak airborne pollen activity in the Irish environment was observed to be bimodal. The first peak period during April–May was dominated by arboreal pollen and the second peak period from June–August was dominated by significantly higher concentrations of herb and grass pollens. Again, this behaviour was mirrored at both monitoring sites. The maximum daily pollen concentration for Dublin was recorded on the 11th of April (614 pollen grains/m^3^) in 2018 and on the 15th of June (15,554 pollen grains/m^3^) in 2019 and the maximum daily pollen concentration for Carlow was recorded on the 13th of June (4854 pollen grains/m^3^) in 2018 and on the 15th of June (20,073 pollen grains/m^3^) in 2019. These peak concentrations were contributed by Poaceae and Urticaceae for those days in June and by *Betula* and *Fraxinus* for the peak concentrations observed in Dublin in April 2018.

Examination of the correlation between the total pollen and major pollen types sampled was used to determine the strength of the association between both sites (Table S1 in Supplemental Information (SI)). Significant correlation was witnessed for *Betula*, Cupressaceae/Taxaceae, *Fraxinus*, *Pinus* and *Plantago* for both years. Whereas, significant positive correlation was only observed for *Alnus*, *Corylus*, *Quercus* and *Rumex* for 2019 and for Poaceae and Total pollen for 2018.

Although the progression of the main pollen types appears similar in both locations, substantial differences between the urban and rural sampling sites were observed. The mean Annual Pollen Integral (APIn) determined for Dublin during the sampling period was 34,217 Pollen × day/m^3^ whereas the mean APIn for Carlow was substantially higher at 78,389 Pollen * day/m^3^. Although the airborne concentrations of pollen appeared lower in Dublin, there were 26 additional pollen types identified in Dublin that were not present in Carlow, many of which were ornamental in nature. The variation in pollen concentration between Dublin and Carlow is mainly attributed to significant Poaceae concentrations in the atmosphere over Carlow during June that is not seen in Dublin. This is depicted in Fig. S1 (SI) where the deviations in average daily pollen concentrations were significantly larger for the month of June. The deviations in pollen concentrations between the two locales do not differ as significantly during the remainder of the documented pollen season.

**Fig. 3:**
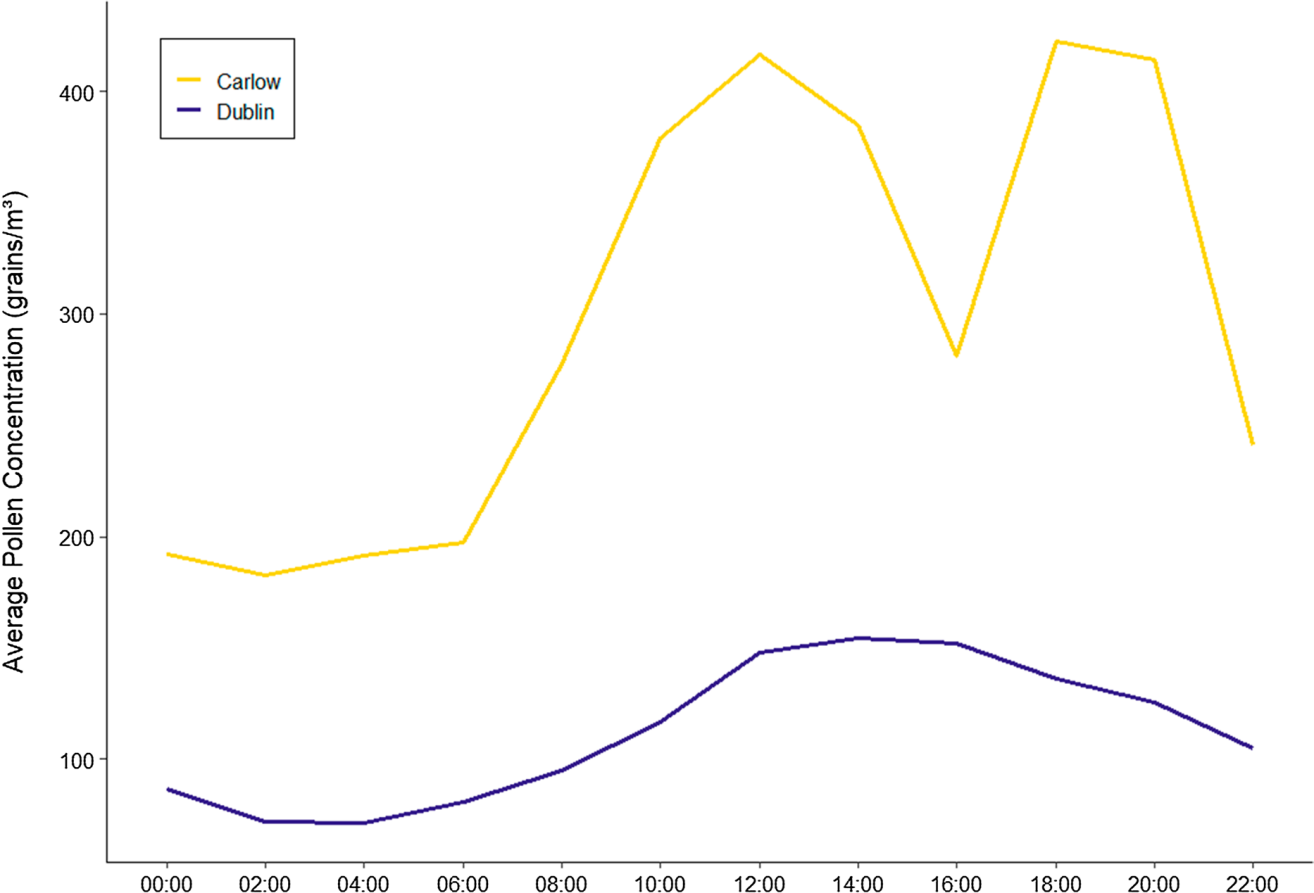
Average hourly distribution of Total Pollen

Additionally, hourly resolution pollen data were analysed in order to determine the hourly peak distributions of pollen concentrations (Fig. 3). Overall, airborne pollen concentrations were shown to peak between 12:00 and 16:00 h for Dublin. However, two peak periods were observed for Carlow, with pollen concentrations peaking at 10:00–14:00 h and again from 18:00 to 20:00 h. Although markedly different, the diurnal distributions of total pollen from both sites are both mainly driven by concentrations of Poaceae pollen, seeing as it was the most prevalent pollen type recorded in both locations. Individual diurnal distributions for *Betula*, Poaceae and Urticaceae are provided in Fig. S2 (SI), the combination of which contributed to 71% and 89% of total pollen sampled at Dublin and Carlow, respectively. In the case of Dublin, Poaceae concentrations reached a single peak concentration at 14:00, with a steady crest being observed from 12:00 to 16:00. For Carlow, two peaks were observed, the first from 10:00 to 14:00, reaching a maximum at 12:00. The second peak arises from 16:00 to 22:00 reaching a maximum at 20:00. In comparison, the diurnal patterns of *Betula* and Urticaceae are not as readily observed from the total pollen trends. *Betula* reaches peak concentrations between 8:00 and 14:00 in Dublin and between 06:00 and 8:00 in Carlow. For Dublin, Urticaceae pollen reaches its peak much later in the evening, between 16:00 and 22:00 whereas a morning peak in Urticaceae between 8:00 and 14:00 is observed in Carlow.

### Meteorological data

Association between seasonal pollen concentrations and individual meteorological factors was also investigated for both locations by calculating Spearman’s rank coefficient between daily pollen concentrations and individual meteorological parameters. Of the pollen types considered, 9 were present at both sites in high concentrations for the duration of the sampling period accounting for an average of 93% and 92% of the total pollen recorded in both Dublin and Carlow, respectively. These pollen types included the three main allergenic pollens associated with seasonal allergies in Ireland, those being: *Alnus, Betula* and Poaceae*,* as well as *Corylus,* Cupressaceae/Taxaceae*, Fraxinus, Quercus, Pinus* and Urticaceae.

The correlation results between individual pollen species and meteorological parameters are presented in Table S2 and Table S3 (SI). It was observed that correlations between pollen types and meteorological parameters produced varying results depending on the year and location. For Dublin, minimum temperature (*T*_min)_, maximum temperature (*T*_max)_ and mean temperature (*T*_mean)_ showed very little significant correlation with any of the tree pollen types or grass pollen during 2018, with the exception of *Quercus* which illustrated a significant negative correlation to all three. Urticaceae also illustrated a negative correlation to *T*_mean_. This behaviour was observed again for Urticaceae in 2019, although significant positive correlations were now observed for the majority of the early season arboreal pollen types (*Alnus, Betula* and *Corylus*) but not for Poaceae or Cupressaceae/Taxaceae. This seemingly capricious trend was observed for the majority of the pollen types and meteorological parameters and was mirrored at the other sampling location.

An increased significant positive correlation was also observed in Carlow for the majority of tree pollens from 2018 to 2019 with temperature, with Urticaceae again maintaining a significant negative correlation for both years. A significant negative correlation between *Alnus* and *Corylus* with wind speed and a significant positive correlation between several of the tree pollen types with soil temperature and potential evapotranspiration was also observed in both locations in 2019. However, significant differences in pollen-weather behaviour were also observed between the two locations. Relative humidity resulted in a significant negative correlation with both tree and grass pollens in Dublin during both sampling years, yet no significant correlation was observed for the same pollen types in Carlow during either season.

Furthermore, little correlation was observed between precipitation amount and pollen concentration. Only 3 pollen types between both sites presented a significant correlation with rain, 2 of which illustrated a significant negative correlation (Cupressaceae/Taxaceae and Urticaceae) with 1 illustrating a positive correlation in 2018 (Poaceae). However, several tree pollen types illustrated significant positive correlations with the average precipitation amount of the previous 10 days in 2018.

A summary of monthly meteorological conditions is also provided in Table S4 (SI), illustrating the varying weather conditions experienced between the two sampling years. It can be seen from Table S4 that during 2018 both sampling locations experienced two meteorological extremes. During February- March, a significant cold spell was experienced with daily temperatures plummeting to − 5 °C. In addition to this, a very dry and hot June was experienced with temperatures reaching highs of 29.8 °C in Carlow and rainfall amounts barely exceeding 5 mm. In comparison, the weather conditions experienced during 2019 were much more stable.

In efforts to provide a more detailed meteorological comparison with the pollen data, monthly Spearman analyses were carried out as shown in Tables S5 and S6 (SI). A strong positive correlation was observed between total pollen and temperature for many of the early months leading up to spring and early summer. Temperature variables in Dublin showed significant positive correlation with total pollen from February until June with a lack of significance observed during July and August which represented the hottest months. An increased correlation between sun duration and global radiation was observed for March, April, June, August, and September. Both of which coincided with peak concentrations of *Betula* and Cupressaceae/Taxaceae pollen during February-April and peak concentrations of Poaceae pollen in June, with sun duration coinciding with peak concentrations of Urticaceae pollen in August–September. Similar trends were observed for total pollen and monthly meteorological trends during the same time in Carlow. However, negative correlation with temperature was observed for total pollen during July in both Carlow and Dublin, mimicking trends seen for Urticaceae pollen. Notable positive correlation was also observed for total pollen with global radiation during April and July during which time, total pollen concentrations were dominated by *Betula* and Poaceae pollen, respectively.

There were several periods when significant correlations were observed for rainfall and relative humidity. A significant negative correlation with rainfall was observed in Dublin during the late summer-autumn period of 2019. During which high amounts of precipitation were observed in comparison to previous years. Similar negative correlations were observed with relative humidity for April, June, and September. Mimicking a similar relationship witnessed by many of the major pollen types from 2019. Negative correlations were also observed for total pollen and relative humidity in Carlow during April, July and September. In addition, a significant decrease in ambient pollen concentrations was noted for March 2019 at both sampling locations due to high rainfall.

In comparison, the monthly weather correlation analysis for total pollen in 2018 was much less consistent. Significant positive correlation between temperature and soil temperature was observed in January of Dublin 2018. However, during February and March of 2018, no correlation could be made between the ambient total pollen concentration and meteorological conditions at either site. Little correlation was observed for April and May and a significant decrease in total pollen (as shown in Tables S7 and S8 (SI)) was observed as several arboreal pollen seasons came to an end. June 2018 represented an extended dry period for both Dublin and Carlow with negative correlation observed for sun and global radiation for Dublin and a notable lack of correlation observed for Carlow. 

### Source -receptor model outputs for Dublin: prevailing wind direction

Wind pollination is an important transport mechanism for a vast range of pollen types (Dowding, 1987) as such the ZeFir source receptor model was used to better understand the transport of airborne pollen grains over Dublin. The resulting wind rose diagrams can provide information on the spatial origins of the pollen. The same approach was recently used to assess the temporal variability and geographical origin of ambient bioaerosols, particularly pollen (Sarda-Estève et al., 2018, 2019, 2020).

Results are depicted by a wind rose plot where sectors are shaded according to the joint probability of the wind originating from that direction. Joint probability provides information on the statistical probability and distribution of prevailing winds during the study period. The white gridlines traversing the wind rose plot represents a wind speed scale in kilometres per hour, the inner circle equating to 2 km/h followed by 4 km/h, 8 km/h and 12 km/h.

**Fig. 4:**
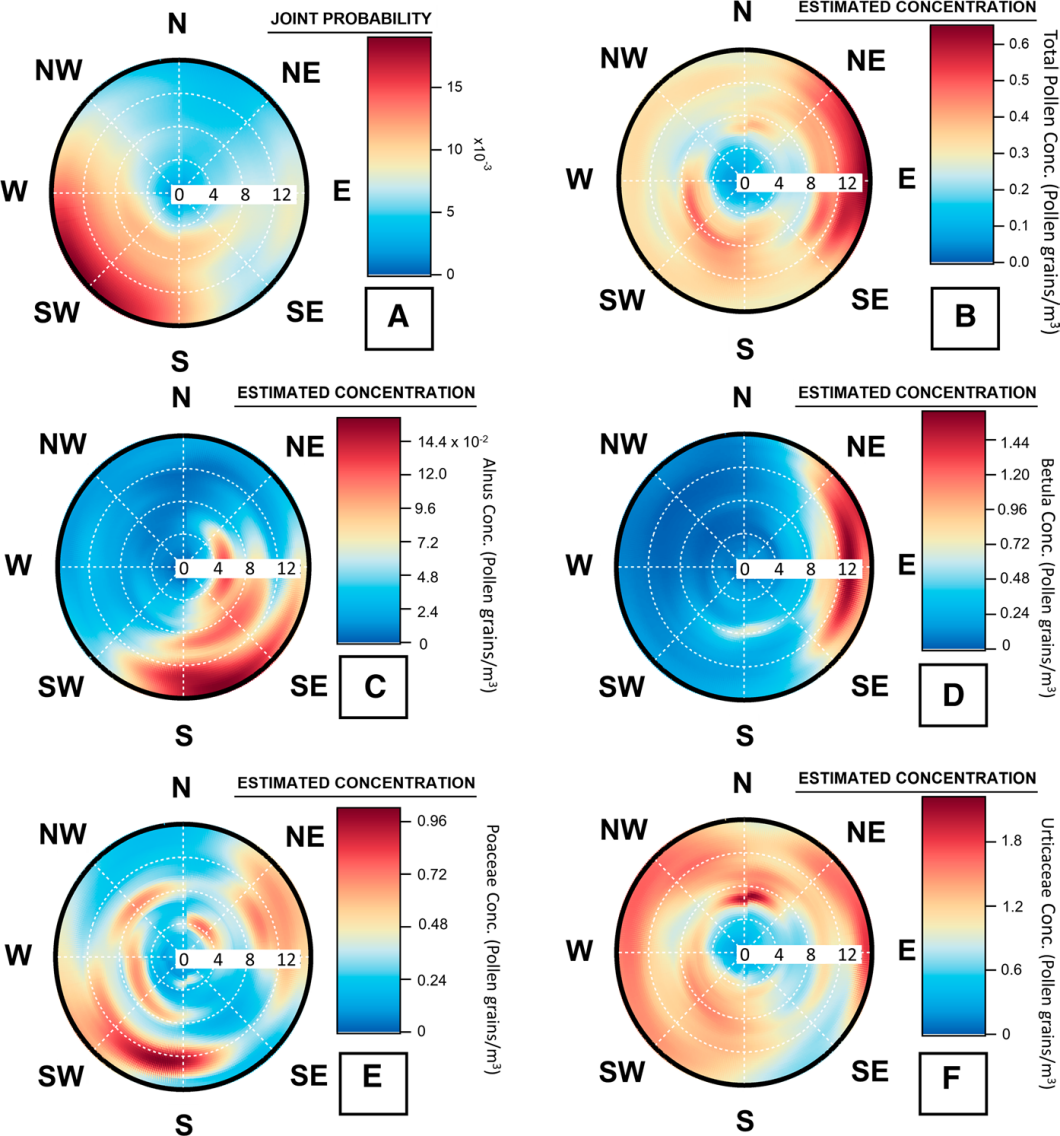
Origin of **A** Prevailing Winds, **B** Total pollen*,* **C Alnus**, **D** *Betula,* **E** Poaceae and **F** Urticaceae pollen at Dublin. The colour scale represents the estimated concentration (Pollen grain/m^3^) white gridlines represent a wind speed scale in kilometer per hour (4 km/h, 8 km/h, 12 km/h)

The model results showed that prevailing winds at Dublin were coming from the Southwest sector at speeds from 4 to over 12 km/h (Fig. 4).

### Source -receptor model outputs: geographical origins

In the case of determining the geographical origin of ambient pollen grains, the resulting wind rose plot is shaded by the estimated particle concentrations (Pollen grains/m^3^) for any wind speed and wind direction. Again, white grid lines represent the wind speed in kilometres per hour (2 km/h, 4 km/h, 8 km/h and 12 km/h).

The spatial origin of total pollen from May 2017 to September 2019 over Dublin was calculated using the SWIM approach. Interestingly, the pollen impacting does not correspond with the main wind direction. Two geographical origins were suggested: the first and the most important one mainly originated from the East sector at speeds of more than 12 km/h indicating long-range transport. The second originating from the Southwest sector at speeds of 8 km/h (Fig. 4). In order to gain a greater insight into the origin of important allergenic pollen types, the SWIM model was also applied to several individual allergenic pollen types from trees (*Alnus* and *Betula*) and herbs (Poaceae and Urticaceae).

As illustrated in Fig. 4, the model results indicate that the main origin for *Alnus* pollen is from a South to South-easterly direction with varying wind speeds from 4 – 12 km/h. Higher concentrations were associated with higher wind speeds from the South. The main origin for *Betula* comes from the East sector and is associates with strong winds up to 12 km/h. For Poaceae, the model shows that high concentrations originate from a southerly direction, linked with strong wind speeds of 12 km/h and to a lesser extent to more moderate wind speeds of less than 8 km/h. The origin of Urticaceae pollen is multidirectional likely due to numerous sources. High concentrations of Urticaceae originated from the East at moderate wind speeds greater than 8 km/h. A second Northern regime also accounts for high concentrations of Urticaceae, associated with wind speeds between 4 and 8 km/h. The only direction for Urticaceae not to originate from is from the Southeast sector. Therefore, the high concentrations of total pollen originating from the East are likely a result of some Urticaceae pollen and high concentrations of *Betula* pollen. The varying wind speeds and directions associated with the differing pollen types provide a greater insight into the geographical origins of different allergenic pollen types, not just total pollen. Knowing the potential origins of airborne pollen can be used to further enhance forecasting applications.

### Pollen calendar—Dublin

**Fig. 5:**
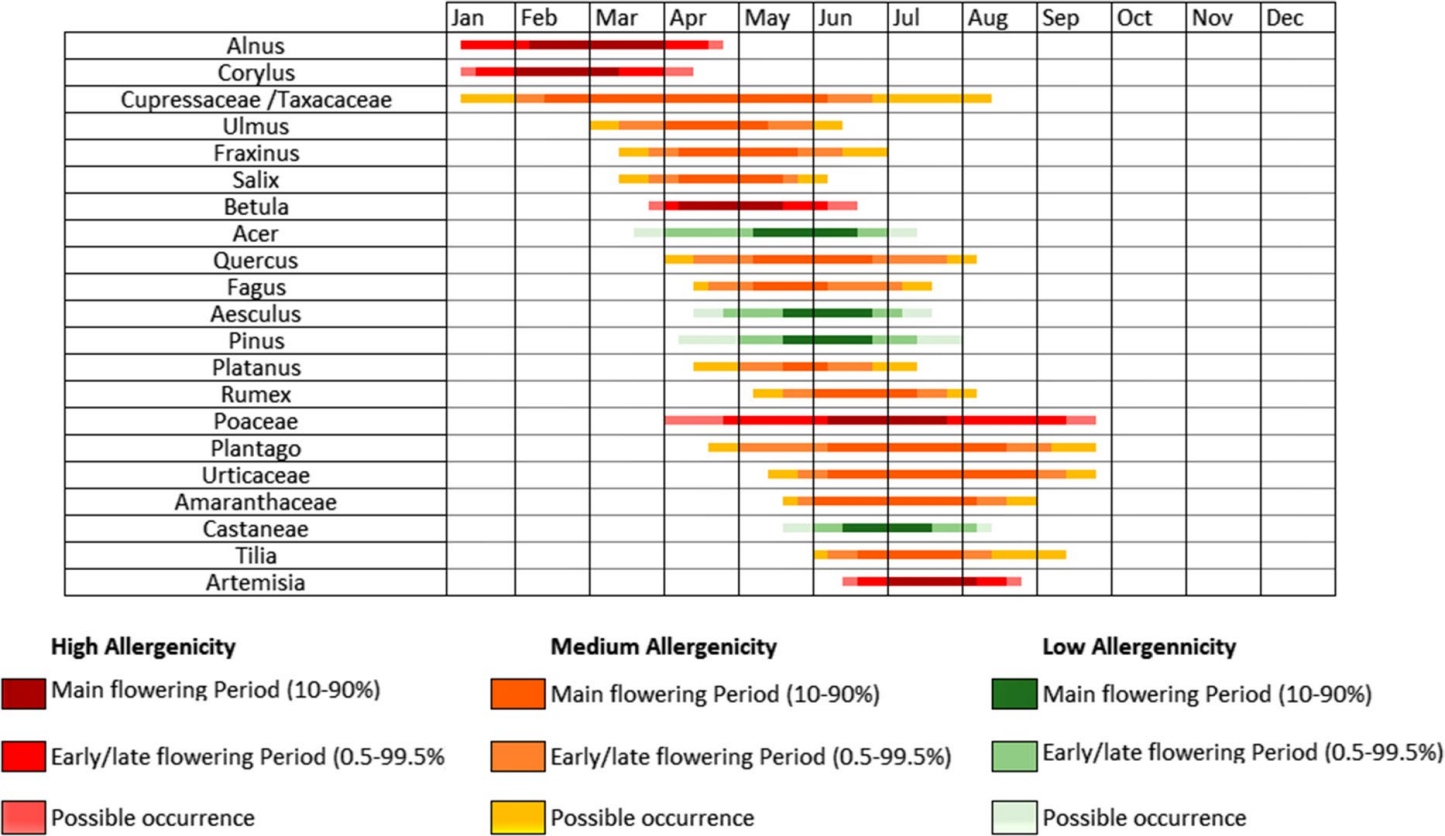
First pollen calendar for Dublin (periods of 1978–1980, 2010–2011 and 2017–2019)

A pollen calendar is a graphical representation of the average annual/seasonal trends of major pollen types, typically those of allergenic concern, for a particular location. Although an approximation of the seasonal trends in Ireland has been presented above, variations exist between each location and its associated pollen season for that year. A start date for the MPS of one year could differ substantially from the next. Therefore, it is recommended that at least 5–7 years of data are incorporated into the construction of a pollen calendar (Galán et al., 2017). For this reason, the data obtained solely from this monitoring campaign is insufficient for the construction of a pollen calendar for either Dublin (2017–2019) or Carlow (2018–2019). Unpublished pollen data from 1978–1980, 2010–2011 was incorporated in creating the first pollen calendar for Dublin (Fig. 5). Whereas additional monitoring is required before a pollen calendar can be constructed for Carlow with any degree of certainty.

Pollen taxa were classified according to their relative allergenic potential as described in the literature (Bousquet et al., 2007; D’Amato et al. 2007a; de Weger et al., 2013; Heinzerling et al., 2005; Pablos et al., 2016; Skjøth et al., 2013). Consideration was also given to general ambient concentrations observed over the sampling years as a factor of relative exposure to the Irish public.

## Discussion

Examination of the recorded pollen data from both sampling locations provided important information about the prevalent pollen types and seasonal trends of the Irish environment. Pollen seasonal trends were similar at both locations and can be described as bimodal, starting in January/February with the releases of *Corylus* and *Alnus* pollen, and ending in late September/early October with the decline of Poaceae and Urticaceae pollen. This observed trend is comparable to aerobiological studies from the United Kingdom (Emberlin et al., 1990, 1993, 2007) and notably different from those recorded in other European regions such as Mediterranean (Camacho, 2015; Galán et al., 1995; Giner et al., 1999; Puljak et al., 2016; Rodríguez et al., 2015; Subiza et al., 1995) and Nordic countries (Nilsson & Persson, 1981; Przedpelska-Wasowicz et al., 2021). The first peak period observed in April and May is due to increased *Betula* and *Fraxinus* concentrations, followed by a second Poaceae-related peak in June and July. The prevalence of *Betula* and Poaceae pollen at both the Dublin and Carlow sites represent the two predominant allergenic pollen types observed during the sampling period, both contributing to notable peak periods in spring and summer, respectively.

This study represents the first comprehensive, multi-season survey of airborne pollen in Ireland. Prior to this, some short-term pollen (McDonald, 1980; McDonald & O’Driscoll, 1980) and Fungal spore (Healy et al., 2014; Martínez-Bracero et al., 2022; O’Connor et al., 2015a, 2015b, 2014a, 2014b) monitoring campaigns were conducted, but none were designed for the continuous or long-term monitoring of pollen trends or for identifying any changes within the network over time. Given that not many previous studies have taken place in Ireland, the few that have been carried out were analysed to identify possible similarities or differences with the current study. The Galway studies of 1977–1978 (McDonald, 1980; McDonald & O’Driscoll, 1980) were of particular note as their data could be directly compared with our own. As the studies were only carried out during the Summer months, complete pollen seasons could not be constructed. Similarities in Poaceae and Urticaceae peak times can be made between the results presented for Galway and those of Dublin and Carlow (McDonald, 1980; McDonald & O’Driscoll, 1980). Results of note include high levels of *Rumex* and *Chenopodium* seen in Galway, these taxa were identified in Dublin during the study period (2017–2019), but only in low concentrations. This indicates that there are regional variations within the country, but this hypothesis needs further investigation as a study of modern pollen trends in Galway is years away.

During the current campaign, marked deviations were observed between the sampling years. Annual pollen concentrations for both sites were higher in 2019 than in 2018, although 2018 had a higher number of major pollen types identified. MPS start times also changed greatly between the two years. Varying factors can influence the ambient concentration of pollen grains in one MPS, these relate to the influences of meteorological conditions on the release, production, dispersal and transportation of pollen (Galán et al., 1995; Volkova et al., 2016). A period of unseasonably cold weather and blizzard-like conditions hit the East coast of Ireland during February and March of 2018, this is the clearest possible reason for the major differences between the two sampling years. The worst conditions were experienced from the 28th of February to the 4th of March with air and ground temperatures 5 to 10° below normal (Government of Ireland and Met Éireann, 2019). Studies have highlighted that early springtime conditions such as temperature are deciding factors for plant development and thus pollen production (Matyasovszky et al., 2015). As a result, the expected release of arboreal pollen in late March was significantly reduced as was the summer release of Poaceae pollen. This corroborates results from similar studies where colder temperatures during early spring were linked to a delay and reduction in arboreal pollen release (Emberlin et al., 2002; Kasprzyk & Borycka, 2019) and a reduction in summertime Poaceae concentrations (Makra et al., 2012). A study by Emberlin et al. (1999) observed a comparable correlation between the soil temperature in early spring and the abundance of grass pollens released during the following summer, corroborating the increased Poaceae pollen concentrations seen for the 2019 season. Overall, the pollen seasonal trends observed during 2018 more accurately mimic those of countries with cooler springtime temperatures such as Nordic countries (Emberlin et al., 2002; Nilsson & Persson, 1981; Przedpelska-Wasowicz et al., 2021).

The stark decrease in *Fraxinus* concentrations in 2019 is the only exception to the increasing trend in pollen abundance seen from 2018 to 2019. This likely resulted due to a combination of various factors. Firstly, during the study period, Ireland was in the midst of an “Ash Dieback” (ADB) epidemic, the presence of which could influence pollen production/release (Teagsc, 2019). The presence of ADB has been shown to reduce *Fraxinus* ambient pollen concentrations (Evans, 2019). However, conflicting reports have also been documented, of an additive effect (Gassner et al., 2019). Other factors such as mast years should also be considered. Many trees experience a variation in reproductive efforts over several years known as masting (Dahl et al., 2013). Studies have found that *Fraxinus* trees have been known to simply experience periods of significantly lower pollen production every couple of years. It is possible that 2019 represents a naturally occurring inactive period within the mast cycle (Gassner et al., 2019) and is not a result of ADB.

The composition of pollen in the atmosphere is dependent on the vegetal cover of the sampled area in conjunction with climatic and weather conditions (Kasprzyk, 2006). As such, it was unsurprising that several differences were noted between the two sampling locations. A direct comparison between the sites showed that the APIn was higher at the rural site (Carlow) and lower at the urban site (Dublin) for both years. The main cause for this stark difference in APIn was attributed to very high Poaceae concentrations observed in Carlow. This difference in Poaceae concentration is most evident in the early summer. A higher concentration of Poaceae for Carlow can be expected as the land use surrounding the sampling point is mainly rural grassland (Fig. 1), whereas the Dublin sampling point is located in an urban setting (Fig. 1). Increased concentrations were also witnessed in Carlow for several other herbaceous pollen types including *Rumex* and *Plantago*, with the exception of Urticaceae. Higher concentrations of Urticaceae pollen were observed in Dublin in 2019 as certain Urticaceae species, namely *Urtica dioca,* are nitrophilous and are often more closely associated with urban sites (Kasprzyk, 2006). Similar trends in grass and herbaceous pollen abundance when comparing urban and rural sites have been documented throughout Europe (Antón et al., 2020; Hugg et al., 2017; Kasprzyk, 2006; Kruczek et al., 2017; Rodríguez-Rajo et al., 2010). There is one other factor that likely influenced the comparison between the two sampling locations: the sampling height of the Hirst traps. The impact of which has been well document in literature (Aulirantio-Lehtimäki et al., 1991; Fernández-Rodríguez et al., 2014; Kolek et al., 2021; Rojo et al., 2019, 2020; Xiao et al., 2013). Many studies corroborate the findings that the concentration of grass and other herbaceous pollen can be several times higher when sampled closer to the ground (Aulirantio-Lehtimäki et al., 1991; Kruczek et al., 2017; Spieksma et al., 2000). It is a combination of these factors that led to a lack of significant correlation between the sites for both total and Poaceae pollen concentrations for 2019 (shown in Table S1).

Certain arboreal pollen types such as *Alnus,* Cupressaceae/Taxaceae and *Quercus* were typically more abundant in the urban location. Again, this behaviour can be partly accredited to the land cover surrounding the sampling site. From examination of the land cover at both sites, it can be observed that the immediate 30 km surrounding the Carlow site is largely dominated by pastures. In comparison, the land cover depicted Dublin is strikingly different, being dominated by various degrees of urban landscapes. However, what is lost in the resolution of the 30 km radius surrounding the Dublin site is the presence of forests and urban green spaces within the city limits. In fact, as of 2018, the total surface occupied by green spaces, broadleaved forests and mixed forests within Dublin city was reported to be twice that of the surface occupied in Carlow (Maya-Manzano et al., 2021). As a result, higher ambient concentrations of certain arboreal pollen types such as *Alnus,* Cupressaceae/Taxaceae and *Quercus* pollen can be expected in Dublin. Uncertainty remains with regards to the concentrations of *Betula* pollen. Higher concentrations of *Betula* pollen were observed in Dublin during 2018 but higher concentrations were observed in Carlow for the 2019 season. There are a number of reasons for this inconsistency; for one, the sampling of the 2018 pollen season in Carlow was initially delayed, potentially missing part of the *Betula* pollination period. Thus, resulting in a lower observed APIn for *Betula*. However, a recent study by Maya-Manzano et al. (2021) noted that several mature birch trees are positioned in relative close proximity to the Carlow sampling trap, which could account for higher than anticipated concentrations seen in 2019.

The increased concentration of tree pollen species as well as the addition of several pollen types not recorded in Carlow (*Platanus*, *Ulmus*, *Fagus*, Ericaceae, *Mercurialis*, *Forsythia*, *Hedera*, *Populus*, Ranunculaceae) can be further explained by the extended use of ornamental plants in urban green spaces which are not usually present in rural settings (Velasco-Jiménez et al., 2020). The presence of certain ornamental trees in Dublin City has been reported in several preceding studies (Ningal et al., 2010; Xie, 2018). Dublin also saw higher concentrations of pollen types associated with coastal and riverside areas such as Juncaceae (El-Amier, 2015; Fall, 1994; Doogue et al., 2004; Fontana, 2005) and the addition of several varieties of herbaceous pollen types, many of which were only observed in small quantities including Ranunculaceae, *Forsythia*, *Sambucus* as well as *Hedera* pollen which likely originated from nearby historical buildings.

Differences between the sites were also observed for average diurnal trends. The diurnal trends in both Dublin and Carlow were primarily driven by Poaceae concentrations. The diurnal plot for Dublin contained one single afternoon-evening peak, which is consistent with diurnal plots found in many studies (Aboulaich et al., 2013; Norris-Hill & Emberlin, 1991; Peel et al., 2014; Spieksma et al., 1985; Yang et al., 2003). Carlow, however, had a diurnal plot with two defined peaks. This morning and nightly peak in Poaceae pollen has been discussed previously (Kosisky et al., 2010; Peel et al., 2014) and can be attributed to early season Poaceae dispersal. Differences in deposition patterns observed in rural areas compared to those in urban areas, with urban settings possessing higher levels of air turbulence (Emberlin & Norris-Hill, 1991a, 1991b), could also explain these regional differences.

This paper depicts the first construction of a pollen calendar for Dublin which graphically represents the pollen trends for individual types of varying allergenicity. Ireland has a high incidence of respiratory disease with the concomitant addition of seasonal allergies. Therefore, this pollen calendar represents a useful resource for allergy sufferers. Several pollen types were classified as having high allergenic concern, including pollen originating from grasses and trees of the *Betulaceae* family which are known for their clinical relevance across Europe (Burbach et al., 2009) and Ireland (Bousquet et al., 2007; Heinzerling et al., 2005). A comparable study by Adams-Groom et al. (2020) recently described a regional pollen calendar for Northern Ireland’s main pollen allergens. This study showed similar trends to that of Dublin, the only difference being that Urticaceae pollen appeared to have a longer pollen season in Northern Ireland (May–September). The similarities in seasonal trends observed between Dublin and Carlow during this investigation, the historical studies from Galway (McDonald, 1980; McDonald & O’Driscoll, 1980) and the pollen calendar from Northern Ireland (Adams-Groom et al., 2020) suggests that general trends for a select few pollen types, irrespective of concentration, appear to be somewhat comparable across Ireland.

Spearman’s correlation coefficient was used to analyse possible correlation trends between meteorological parameters and pollen concentration data obtained throughout the study period. The lack of correlation seen in Dublin during early spring of 2018 was attributed to adverse weather conditions, leading to inconsistencies and delays in springtime pollen release, as previously discussed. The correlation analysis identified a positive correlation between air temperature and proliferation of arboreal pollen such as for *Alnus*, *Betula*, *Corylus* in Dublin and *Quercus* in Carlow. The importance of temperature on pollen production is well established in the literature (Emberlin et al., 1993; Frenguelli et al., 1991; Khwarahm et al., 2014; Norris-Hill, 1997). These arboreal pollens also negatively correlated with relative humidity coinciding with similar findings found in previous studies (Pérez-Badia et al., 2011; Puc, 2012). The monthly spearman analysis between total pollen and meteorological parameters showcases the importance of certain meteorological parameters pre and post-major pollen peak dates. Strong association with temperature was seen for January 2018. Temperatures at the start of the year have been shown to play an important role in initiating the onset of the pollen season for spring flowering plants (Emberlin et al., 2002). As such, this strong positive correlation is representative of pre-peak season correlations seen in other studies (Khwarahm et al., 2014; Piotrowska & Kaszewski, 2012).

Conversely, a negative correlation was observed between *Fraxinus* pollen and temperature in 2018 in Dublin. A similar trend was also observed for the total pollen and temperature in Carlow in April 2018. Although many tree pollen types have been shown to possess a generally positive correlation with temperature, *Fraxinus* pollen has been shown to illustrate a negative association (Álvarez-López et al., 2020). A significant negative relationship with temperature was also seen for Urticaceae pollen, from Carlow 2019, likely depicting the effects of heat stress on pollen release (Emberlin & Norris-Hill, 1991b). From further examination of the monthly correlation, a notable decline in temperature was noted following June/July when most pollen types were in decline. This supports previous findings that temperature dependence decreases following peak pollen season (Khwarahm et al., 2014). In 2018 a notable increase in temperature dependence was seen in September during which most pollen types were in steady decline, representing a period of resuspension (Rojo et al., 2020).

Sun duration and global radiation were also observed to positively impact many arboreal and herbaceous pollen taxa, particularly during 2019. The importance of sun duration and global radiation for pollen release and production has been highlighted in the literature (Gioulekas et al., 2004; González-Fernández et al., 2021; Khwarahm et al., 2014). This is particularly true for grass and herb pollen types (Çakir & Doğan, 2020; de La Guardia et al., 1998; Myszkowska, 2012). A notable negative association with sunshine was observed for Poaceae pollen in Dublin during 2018 with a notable lack of correlation observed for Carlow. This is largely due to the extremely dry conditions witnessed during much of May and the month of June 2018. Such dry conditions have been shown to reduce grass growth and pollen production (González Minero et al., 1998), thus accounting for the lack of correlation between Poaceae pollen and temperature and the negative correlation between total pollen and temperature during June 2018 (Dublin). Many grass species are dependent on precipitation and have a low tolerance to drought (Jung et al., 2021), explaining the positive correlation observed between Poaceae pollen and rainfall in Carlow 2018.

Negative correlations with rainfall were not commonly witnessed for major pollen types. The negative correlations that were observed were caused by particle-deposition driven by washout processes (Kluska et al., 2020) as seen extensively throughout literature (Bruffaerts et al., 2018; Kluska et al., 2020; Ribeiro et al., 2003). When examining the monthly correlation of total pollen with rainfall, a primarily negative correlation was seen, suggesting that rainfall attenuates total pollen throughout the entire season. However, several major pollen types indicated a positive correlation with the average rainfall of the previous 10 days, meaning that rainy days prior to pollen release may trigger flower blooming and pollen release (Khwarahm et al., 2014). A notable positive correlation was noted between rainfall and total pollen during January 2019, when concentrations of *Corylus* and *Alnus* pollen had yet to reach their peak, illustrating the link between rainfall and the opening of anthers (Newnham et al., 2013).

Both positive and negative correlations were noted between wind speed and several major pollen types/monthly pollen concentrations. Windspeed has long been identified as a means by which pollen is readily released from catkin (Sofiev et al., 2013). However, wind speed is also connected to the atmospheric transport of pollen, with positive correlation indicating the transport of pollen from outside the sampling area and negative correlation suggesting transport of pollen from the immediate area. To further evaluate the effects wind speed and direction had on the transportation and spatiotemporal variation of observed pollen concentration, the geographical origins of total and specific pollen types were investigated for Dublin. From examination of Fig. 4, it can be observed that possible sources of long- and short-range transport varied by pollen type. *Betula* pollen was seen to be originating from a prevailing Easterly direction at high wind speeds, corroborating similar findings by Maya-Manzano et al. (2021). High concentrations of Poaceae and *Alnus* pollen appeared to originate from a south-westerly and south-easterly direction, respectively. It is possible that this pollen originated from various forests and pastures located south of Dublin city or from other major sources like the Wicklow Mountains National Park (south of Dublin), as previously indicated by Maya-Manzano et al. (2021).

Although several significant correlations were noted for various pollen types and meteorological parameters, a strong degree of variance was witnessed depending on the year, sampling site and pollen type in question. Compared to other European studies (Hoebeke et al., 2018), monitoring efforts in Ireland are very much in their infancy. Therefore, it is likely that in years to come, more apparent trends will come to light with regard to such analyses.

## Conclusion

This study illustrates the findings of the first comprehensive pollen survey of Ireland. The additional availability of historical (unpublished) data from 1978–1980 and 2010–2011 also allowed the first Dublin pollen calendar to be developed. The aerobiological monitoring of pollen concentrations at two sampling sites (one urban, one rural) illustrated that the pollen season in Ireland is largely bi-modal, commencing in January/February with the release of arboreal pollen such as *Corylus* and *Alnus*. A peak period is observed in spring (March–April) due to high concentrations of *Betula* pollen and is later followed by a second, more intense peak period during summer (June-July) due to high ambient concentrations of Poaceae and Urticaceae pollen. The pollen season then begins to decline, coming to an end in late September. Prevalent pollen types for Dublin were determined to be Poaceae, Urticaceae, Cupressaceae/Taxaceae, *Betula, Quercus*, *Pinus*, *Fraxinus* and *Alnus.* Comparable prevalent pollen types were identified for Carlow: Poaceae, Urticaceae, *Betula, Quercus, Fraxinus* and *Pinus*. In terms of annual trends, an increase in APIn was observed for most major pollen types apart from *Fraxinus* which saw a decrease in concentration in 2019.

Several deviations between the urban and rural sites were also noted. Overall, the Carlow site exhibited higher annual pollen concentrations, largely driven by Poaceae concentrations. Conversely, the urban site exhibited a greater range of pollen types due to the increased presence of ornamental plants/trees, such as *Platanus*, *Ulmus*, *Fagus*, Ericaceae, *Quercus* and Cupressaceae/Taxaceae. Additional herbaceous pollens were also observed at the Dublin site originating from urban gardens and green spaces.

Correlation analysis between meteorological parameters and ambient pollen concentrations using Spearman rank correlation illustrated varying results depending on the month, year and location. Several early arboreal pollen types showed a positive correlation with air temperature and illustrated negative correlations with relative humidity. Apart from this, few consistent trends were observed for the correlation between pollen concentrations and meteorological parameters. This was attributed to the abnormally cold weather experienced during early 2018 and very dry and hot conditions experienced during the summer of 2018. These conditions resulted in the reduction of arboreal pollen released during early March and influenced the later production and release of Poaceae. Increased monitoring efforts are thus required to significantly demonstrate reoccurring annual and seasonal trends with meteorological parameters. Further analysis of the effects of wind direction and speed on selected allergenic pollen types for Dublin yielded information regarding their geographical origin. Poaceae pollen was seen to originate from a southwest direction, whereas *Alnus* and *Betula* pollen originated from the Southeast and East, indicating potential transport from the UK.

## Supplementary Information

Below is the link to the electronic supplementary material.

Supplementary file1 (TIFF 2051 KB)

Supplementary file2 (TIFF 2344 KB)

Supplementary file3 (DOCX 14 KB)

Supplementary file4 (DOCX 21 KB)

Supplementary file5 (DOCX 21 KB)

Supplementary file6 (DOCX 20 KB)

Supplementary file7 (DOCX 20 KB)

Supplementary file8 (DOCX 20 KB)

Supplementary file9 (DOCX 18 KB)

Supplementary file10 (DOCX 17 KB)
