# Supplementary figures and images for: A comprehensive aerobiological study of the airborne pollen in the Irish environment
Source: Aerobiologia (Bologna). 2022 Jul 28;38(3):343–66. doi: 10.1007/s10453-022-09751-w (PMC9526691; doi:10.1007/s10453-022-09751-w)

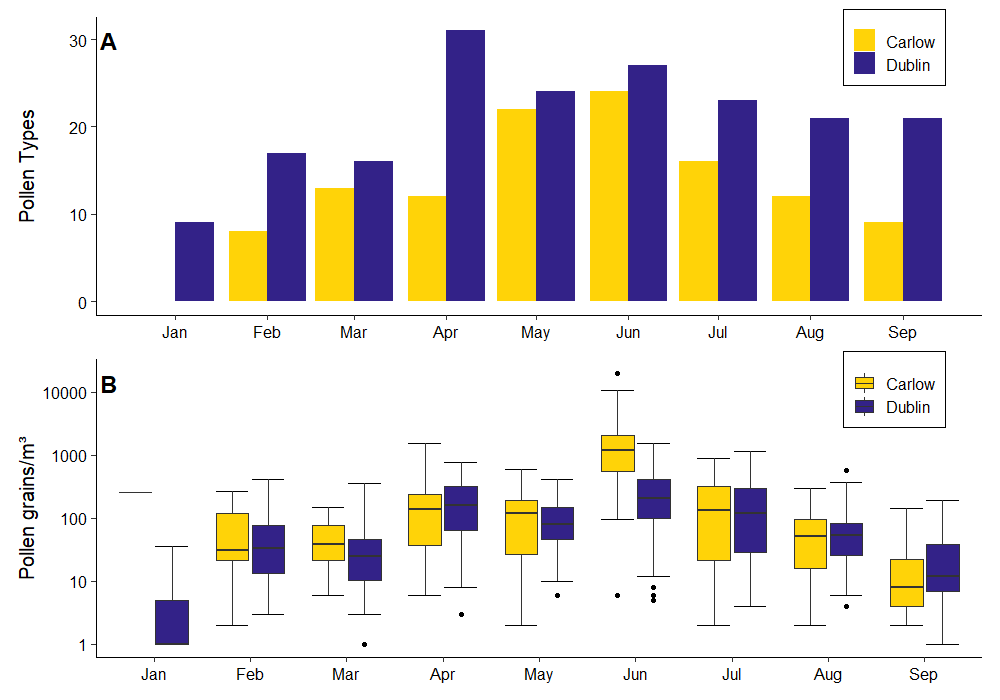

Supplement: Supplementary file 1 — Supplementary file1 (TIFF 2051 KB) [file 10453_2022_9751_MOESM1_ESM.tiff]

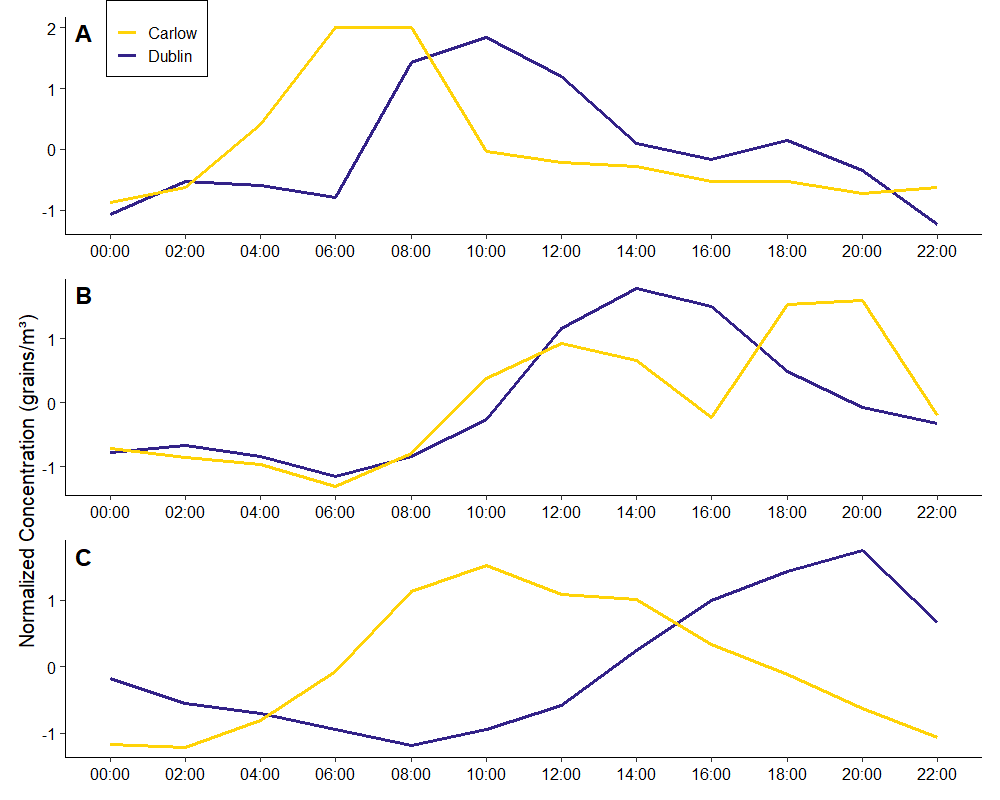

Supplement: Supplementary file 2 — Supplementary file2 (TIFF 2344 KB) [file 10453_2022_9751_MOESM2_ESM.tiff]
