# Supplementary material for: A comprehensive aerobiological study of the airborne pollen in the Irish environment
Source: Aerobiologia (Bologna). 2022 Jul 28;38(3):343–66. doi: 10.1007/s10453-022-09751-w (PMC9526691; doi:10.1007/s10453-022-09751-w)
Supplement: Supplementary file 3 — Supplementary file3 (DOCX 14 KB) [file 10453_2022_9751_MOESM3_ESM.docx]

| Major Pollen | 2018 | 2019 |
| --- | --- | --- |
| *Alnus* | -0.01 | **0.64**** |
| *Betula* | **0.58*** | **0.72**** |
| *Corylus* | -0.03 | **0.57**** |
| Cupressaceae/Taxaceae | **0.42**** | **0.49**** |
| *Fraxinus* | **0.49**** | **0.65**** |
| *Pinus* | **0.48**** | **0.47**** |
| *Plantago* | **0.51**** | **0.6**** |
| Poaceae | **0.68**** | 0.61 |
| *Quercus* | 0.31 | **0.61**** |
| *Rumex* | 0.09 | **0.5**** |
| Urticaceae | **0.71**** | **0.61**** |
| Total Pollen | **0.79**** | 0.41 |

**Table S1** Spearman correlations between pollen concentrations in Carlow and Dublin

**significance at the 95% level, **significance at the 99% level*
