# Supplementary material for: A comprehensive aerobiological study of the airborne pollen in the Irish environment
Source: Aerobiologia (Bologna). 2022 Jul 28;38(3):343–66. doi: 10.1007/s10453-022-09751-w (PMC9526691; doi:10.1007/s10453-022-09751-w)
Supplement: Supplementary file 4 — Supplementary file4 (DOCX 21 KB) [file 10453_2022_9751_MOESM4_ESM.docx]

| Dublin 2018 | | | | | | | | | |
| --- | --- | --- | --- | --- | --- | --- | --- | --- | --- |
|  | *Alnus* | *Betula* | *Corylus* | Cupressaceae/  Taxaceae | *Fraxinus* | *Pinus* | Poaceae | *Quercus* | Urticaceae |
| T_max_ | -0.13 | -0.15 | 0.13 | 0.02 | **-0.44*** | 0.21 | -0.05 | **-0.52**** | -0.23 |
| T_min_ | -0.05 | 0.24 | 0.06 | 0.12 | -0.03 | -0.24 | 0.23 | **-0.63**** | -0.05 |
| T_mean_ | -0.1 | -0.02 | 0.13 | 0.06 | -0.36 | 0.1 | -0.07 | **-0.67**** | **-0.23*** |
| T_mean_10_ | 0.04 | -32 | 0.07 | 0.05 | -0.38 | 0.28 | 0.04 | **-0.71**** | **-0.61**** |
| Gmin | 0.09 | 0.15 | 0.2 | 0.12 | 0.04 | -0.21 | 0.25 | **-0.53**** | 0.01 |
| Rain | -0.06 | 0.11 | 0.02 | 0.03 | 0.16 | -0.19 | 0.15 | 0.15 | -0.03 |
| Rain_10 | -0.05 | **0.4**** | 0.14 | **0.04**** | **0.48*** | -0.26 | -0.01 | **0.64**** | **-0.17*** |
| Wind_S | **0.37*** | 0.21 | 0.08 | -0.02 | -0.19 | -0.32 | **0.26**** | 0.16 | **0.15**** |
| Wind_D | -0.21 | -0.35 | 0.19 | 0.1 | **-0.66**** | **-0.5*** | **0.44**** | -0.01 | -0.01 |
| Sun | -0.12 | -0.18 | 0.06 | -0.19 | **-0.57*** | 0.13 | **-0.24*** | -0.02 | -0.03 |
| G_rad | -0.04 | -0.17 | -0.05 | -0.07 | **-0.64**** | 0.21 | -0.21 | -0.08 | 0.04 |
| Soil | -0.14 | -0.16 | 0.13 | 0.05 | -0.44 | **0.4*** | -0.15 | **-0.66**** | -0.11 |
| Pe | 0.19 | -0.12 | -0.01 | 0 | **-0.52*** | 0.25 | -0.07 | **-0.26*** | -0.02 |
| Evap | 0.24 | -0.06 | -0.03 | 0 | **-0.55**** | 0.2 | 0 | -0.21 | 0.03 |
| Rh | -0.23 | 0.11 | -0.06 | 0.05 | **0.53**** | **-0.11**** | -0.15 | 0.18 | 0.15 |
| Cbl | 0.11 | -0.25 | 0.02 | -0.03 | -0.29 | **0.43**** | **-0.34**** | -0.11 | -0.06 |
| Dublin 2019 | | | | | | | | | |
|  | *Alnus* | *Betula* | *Corylus* | Cupressaceae/  Taxaceae | *Fraxinus* | *Pinus* | Poaceae | *Quercus* | Urticaceae |
| T_max_ | **0.73**** | **0.29**** | **0.5**** | 0.11 | **0.34**** | 0.05 | 0.19 | -0.15 | -0.16 |
| T_min_ | 0.45 | -0.05 | 0.21 | **-0.14*** | -0.02 | -0.18 | -0.02 | -0.14 | **-0.34*** |
| T_mean_ | **0.66**** | **0.21**** | **0.4*** | -0.01 | **0.21*** | -0.06 | 0.1 | -0.16 | -0.3 |
| T_mean_10_ | **0.42*** | -0.36 | 0.23 | **-0.25**** | -0.22 | **-0.47**** | **-0.22*** | -0.33 | **-0.44**** |
| Gmin | 0.39 | -0.17 | 0.27 | **-0.26**** | -0.16 | -0.18 | -0.04 | -0.14 | -0.25 |
| Rain | -0.33 | -0.3 | -0.02 | **-0.29*** | -0.14 | -0.27 | -0.21 | -0.01 | -0.36 |
| Rain_10 | 0.4 | -0.02 | 0.24 | 0 | -0.23 | **-0.45**** | -0.37 | 0.04 | -0.03 |
| Wind_S | -0.03 | -0.03 | 0.14 | -0.11 | -0.19 | -0.17 | **-0.33*** | 0.06 | **-0.31*** |
| Wind_D | **-0.51*** | 0.08 | **-0.31*** | -0.05 | -0.12 | 0.14 | 0.06 | 0 | -0.04 |
| Sun | 0.02 | **0.4**** | 0.05 | 0.07 | **0.21*** | 0.05 | 0.26 | 0 | 0.14 |
| G_rad | **0.26*** | **0.3*** | 0.19 | -0.11 | **0.28*** | 0.06 | 0.32 | 0.03 | 0.26 |
| Soil | **0.62**** | -0.02 | **0.41**** | **-0.1*** | **0.21*** | 0.01 | 0.17 | -0.25 | -0.06 |
| Pe | **0.56**** | **0.31**** | **0.37**** | **-0.09*** | **0.34**** | 0.02 | 0.28 | -0.01 | 0.18 |
| Evap | **0.61**** | **0.32*** | **0.41**** | **-0.12*** | **0.34**** | 0.03 | 0.28 | 0.03 | 0.21 |
| Rh | **-0.54**** | **-0.35*** | **-0.5**** | -0.13 | -0.18 | -0.17 | **-0.31*** | -0.16 | **-0.23*** |
| Cbl | **0.62**** | **0.25*** | **0.4*** | **0.23**** | -0.1 | **0.32*** | **0.42*** | -0.26 | **0.58**** |

**Table S2** Spearman´s rank correlation coefficients between daily MPS 2018 & 2019 Dublin pollen data and meteorological parameters

**significance at the 95% level, **significance at the 99% level*
