# Supplementary material for: A comprehensive aerobiological study of the airborne pollen in the Irish environment
Source: Aerobiologia (Bologna). 2022 Jul 28;38(3):343–66. doi: 10.1007/s10453-022-09751-w (PMC9526691; doi:10.1007/s10453-022-09751-w)
Supplement: Supplementary file 5 — Supplementary file5 (DOCX 21 KB) [file 10453_2022_9751_MOESM5_ESM.docx]

| Carlow 2018 | | | | | | | | | |
| --- | --- | --- | --- | --- | --- | --- | --- | --- | --- |
|  | *Alnus* | *Betula* | *Corylus* | Cupressaceae/  Taxaceae | *Fraxinus* | *Pinus* | Poaceae | *Quercus* | Urticaceae |
| T_max_ | - | -0.31 | - | -0.11 | -0.14 | 0.2 | **-0.44*** | 0.01 | **0.21*** |
| T_min_ | - | -0.25 | - | **-0.22**** | -0.26 | -0.03 | -0.12 | -0.38 | **-0.3*** |
| T_mean_ | - | -0.31 | - | **-0.17**** | -0.31 | 0.16 | **-0.43*** | -0.12 | 0.03 |
| T_mean_10_ | - | **-0.59*** | - | **-0.25**** | 0.23 | **-0.38*** | -0.26 | **-0.38**** | **-0.52**** |
| Gmin | - | -0.2 | - | **-0.18*** | -0.1 | -0.11 | -0.13 | -0.31 | **-0.33**** |
| Rain | - | 0.11 | - | 0.07 | -0.2 | -0.28 | **0.23*** | -0.02 | **-0.33*** |
| Rain_10 | - | 0.5 | - | -0.01 | 0.15 | **0.61**** | 0.12 | **0.41**** | **-0.42**** |
| Wind_S | - | 0.44 | - | -0.16 | -0.54 | -0.3 | -0.01 | -0.03 | **-0.23*** |
| Wind_D | - | 0.19 | - | **-0.21**** | **-0.24*** | -0.43 | 0.07 | **-0.35**** | **-0.35**** |
| G_rad | - | 0.03 | - | 0.15 | -0.17 | 0.3 | -0.3 | 0.24 | **0.52**** |
| Soil | - | -0.38 | - | -0.06 | -0.2 | 0.09 | -0.41 | -0.05 | **0.38**** |
| Pe | - | -0.1 | - | 0.04 | -0.24 | 0.28 | **-0.4*** | 0.13 | **0.46**** |
| Evap | - | -0.06 | - | 0.07 | -0.32 | 0.25 | **-0.4*** | 0.18 | **0.48**** |
| Rh | - | 0.05 | - | 0.05 | -0.18 | 0.17 | **0.45*** | -0.06 | **-0.33**** |
| Cbl | - | -0.25 | - | 0 | 0.29 | 0.3 | -0.17 | 0.17 | **0.39**** |
| Carlow 2019 | | | | | | | | | |
|  | *Alnus* | *Betula* | *Corylus* | *Cupressaceae/*  *Taxaceae* | *Fraxinus* | *Pinus* | *Poaceae* | *Quercus* | *Urticaceae* |
| T_max_ | **0.42**** | **0.51**** | 0.11 | 0.07 | **0.32**** | 0.48 | 0.24 | **0.44*** | **-0.5**** |
| T_min_ | 0.01 | -0.11 | -0.15 | **-0.22*** | -0.02 | 0.16 | 0.23 | 0.25 | **-0.62**** |
| T_mean_ | **0.22**** | **0.4*** | -0.03 | -0.06 | **0.19*** | 0.4 | 0.28 | 0.41 | **-0.59**** |
| T_mean_10_ | **0.4**** | -0.22 | -0.01 | **-0.37**** | -0.09 | -0.12 | 0.19 | 0.09 | **-0.63**** |
| Gmin | 0.04 | -0.39 | -0.06 | **-0.26*** | -0.11 | 0.13 | 0.21 | 0.15 | **-0.57**** |
| Rain | -0.09 | -0.32 | -0.11 | -0.15 | -0.3 | -0.23 | -0.2 | -0.14 | -0.16 |
| Rain_10 | **-0.28*** | -0.15 | **-0.49**** | -0.12 | -0.19 | -0.42 | **-0.63*** | 0.04 | 0.18 |
| Wind_S | -0.21 | -0.19 | -0.12 | 0.12 | -0.14 | -0.17 | -0.08 | 0.07 | -0.16 |
| Wind_D | **-0.09*** | -0.12 | **-0.22*** | -0.19 | -0.09 | **0.41*** | -0.06 | 0.19 | -0.02 |
| G_rad | 0.22 | **0.46**** | -0.12 | 0.13 | 0.16 | 0.18 | 0.02 | 0.24 | 0.16 |
| Soil | **0.28*** | **0.35*** | -0.09 | -0.19 | 0.09 | 0.41 | 0.32 | 0.31 | **-0.44**** |
| Pe | **0.26*** | **0.48**** | -0.23 | 0.02 | 0.17 | 0.35 | 0.08 | **0.34*** | -0.09 |
| Evap | 0.24 | **0.45*** | -0.27 | 0.04 | 0.15 | 0.28 | 0.07 | 0.32 | -0.06 |
| Rh | -0.02 | -0.33 | 0.03 | -0.1 | -0.18 | -0.22 | -0.16 | -0.21 | -0.12 |
| Cbl | 0.13 | 0.03 | -0.03 | 0.12 | 0.16 | -0.22 | 0.13 | -0.21 | 0.09 |

**Table S3** Spearman´s rank correlation coefficients between daily MPS 2018 & 2019 Carlow pollen data and meteorological parameters

**significance at the 95% level, **significance at the 99% level*
