# Supplementary material for: A comprehensive aerobiological study of the airborne pollen in the Irish environment
Source: Aerobiologia (Bologna). 2022 Jul 28;38(3):343–66. doi: 10.1007/s10453-022-09751-w (PMC9526691; doi:10.1007/s10453-022-09751-w)
Supplement: Supplementary file 6 — Supplementary file6 (DOCX 20 KB) [file 10453_2022_9751_MOESM6_ESM.docx]

| Dublin 2018 | | | | | | | | | Dublin 2019 | | | | | | |
| --- | --- | --- | --- | --- | --- | --- | --- | --- | --- | --- | --- | --- | --- | --- | --- |
|  | T_mean_ | T_max_ | T_min_ | Rain | | Wind_S | G_rad | Soil | T_mean_ | T_max_ | T_min_ | Rain | Wind_S | G_rad | Soil |
| January | 5.30 | 13.10 | -3.20 | 93.10 | | 14.80 | 241.13 | 4.40 | 5.10 | 11.30 | -5.80 | 26.80 | 9.30 | 219.16 | 5.18 |
| February | 3.40 | 12.00 | -4.90 | 36.90 | | 11.90 | 523.39 | 3.47 | 7.00 | 15.60 | -3.80 | 30.50 | 10.20 | 541.86 | 6.08 |
| March | 4.30 | 11.90 | -5.10 | 100.00 | | 12.20 | 698.68 | 3.76 | 7.30 | 16.80 | -2.10 | 92.50 | 11.30 | 920.68 | 7.27 |
| April | 8.10 | 18.80 | -2.10 | 68.90 | | 10.80 | 1209.80 | 8.84 | 8.00 | 21.70 | -2.00 | 74.60 | 9.30 | 1277.92 | 9.39 |
| May | 11.40 | 22.20 | 0.40 | 19.10 | | 8.80 | 1835.48 | 14.57 | 10.20 | 20.90 | -0.80 | 33.40 | 7.80 | 1559.48 | 12.81 |
| June | 14.50 | 26.50 | 3.80 | 4.80 | | 8.70 | 2163.20 | 19.82 | 12.50 | 22.70 | 2.00 | 82.90 | 8.20 | 1729.33 | 14.98 |
| July | 16.10 | 26.70 | 5.20 | 40.00 | | 6.90 | 1688.39 | 20.23 | 15.90 | 24.90 | 4.40 | 41.00 | 8.00 | 1722.16 | 18.94 |
| August | 15.30 | 25.10 | 3.90 | 48.00 | | 8.10 | 1290.16 | 16.88 | 15.40 | 22.30 | 7.80 | 91.90 | 8.80 | 1457.26 | 16.69 |
| September | 12.20 | 23.00 | 0.40 | 43.80 | | 9.00 | 1072.50 | 13.37 | 13.00 | 20.90 | 3.40 | 104.60 | 8.80 | 1066.80 | 14.20 |
| Carlow 2018 | | | | | | | | | Carlow 2019 | | | | | | |
|  | T_mean_ | T_max_ | T_min_ | | Rain | Wind_S | G_rad | Soil | T_mean_ | T_max_ | T_min_ | Rain | Wind_S | G_rad | Soil |
| January | 5.40 | 13.30 | -4.70 | 108.10 | | 9.70 | 270.68 | 4.57 | 5.90 | 11.90 | -3.70 | 30.90 | 6.40 | 246.52 | 5.97 |
| February | 3.60 | 13.40 | -5.20 | 38.70 | | 7.70 | 570.00 | 3.71 | 7.50 | 15.10 | -2.30 | 36.80 | 10.10 | 529.36 | 6.57 |
| March | 4.80 | 12.80 | -5.40 | 98.10 | | 7.40 | 763.45 | 5.44 | 7.40 | 16.00 | -0.30 | 122.90 | 9.30 | 943.68 | 7.79 |
| April | 9.00 | 19.00 | -2.70 | 73.00 | | 8.20 | 1286.60 | 10.24 | 8.90 | 22.90 | -1.20 | 72.50 | 7.30 | 1177.77 | 10.35 |
| May | 12.50 | 23.60 | 2.80 | 24.30 | | 6.70 | 1835.26 | 15.44 | 11.00 | 20.90 | -0.50 | 14.10 | 5.90 | 1684.42 | 14.44 |
| June | 16.40 | 29.80 | 5.50 | 5.20 | | 5.60 | 2178.60 | 21.22 | 13.00 | 25.00 | 2.70 | 55.00 | 6.60 | 1753.57 | 16.16 |
| July | 17.80 | 27.90 | 8.60 | 42.50 | | 5.90 | 1841.48 | 21.62 | 16.70 | 25.00 | 6.90 | 42.60 | 6.40 | 1740.97 | 19.52 |
| August | 16.00 | 26.60 | 6.20 | 39.80 | | 7.10 | 1314.55 | 17.79 | 16.20 | 25.10 | 6.70 | 86.40 | 8.20 | 1422.71 | 17.27 |
| September | 12.80 | 23.40 | 0.40 | 53.70 | | 7.50 | 1088.83 | 14.17 | 13.70 | 21.80 | 4.20 | 116.70 | 6.90 | 1076.87 | 15.20 |

**Table S4** Descriptive monthly summary of meteorological parameters
