# Supplementary material for: A comprehensive aerobiological study of the airborne pollen in the Irish environment
Source: Aerobiologia (Bologna). 2022 Jul 28;38(3):343–66. doi: 10.1007/s10453-022-09751-w (PMC9526691; doi:10.1007/s10453-022-09751-w)
Supplement: Supplementary file 7 — Supplementary file7 (DOCX 20 KB) [file 10453_2022_9751_MOESM7_ESM.docx]

| Dublin 2018 - Total Pollen | | | | | | | | | |
| --- | --- | --- | --- | --- | --- | --- | --- | --- | --- |
|  | January | February | March | April | May | June | July | August | September |
| T_max_ | **0.72**** | 0.11 | -0.42 | 0.07 | 0.27 | -0.24 | 0.34 | 0.23 | **0.35*** |
| T_min_ | **0.52*** | 0.08 | -0.07 | 0.37 | **0.27*** | -0.09 | -0.26 | 0.27 | 0.06 |
| T_mean_ | **0.63**** | 0.10 | -0.21 | 0.15 | 0.35 | -0.30 | 0.02 | 0.35 | 0.25 |
| T_mean_10_ | -0.32 | -0.15 | 0.78 | -0.15 | -0.13 | -0.13 | **-0.28*** | -0.15 | 0.19 |
| Gmin | 0.23 | 0.28 | 0.02 | 0.27 | 0.05 | 0.21 | -0.34 | 0.16 | 0.06 |
| Rain | -0.14 | -0.22 | -0.01 | 0.14 | -0.31 | 0.48 | -0.28 | 0.02 | 0.13 |
| Rain_10 | 0.00 | -0.27 | -0.14 | **0.30*** | -0.19 | 0.37 | -0.47 | **-0.46*** | 0.03 |
| Wind_S | 0.43 | 0.02 | 0.05 | 0.01 | -0.38 | 0.24 | 0.15 | 0.28 | 0.13 |
| Wind_D | 0.56 | -0.24 | -0.40 | -0.30 | -0.18 | **0.34*** | **-0.34*** | -0.14 | 0.26 |
| Sun | 0.06 | -0.08 | 0.04 | -0.31 | -0.07 | **-0.48*** | **0.59**** | -0.15 | 0.27 |
| G_rad | 0.08 | 0.02 | 0.14 | -0.29 | -0.08 | **-0.50*** | **0.62**** | -0.17 | **0.36*** |
| Soil | **0.60**** | -0.06 | 0.28 | 0.04 | 0.34 | **-0.63**** | **0.61*** | 0.26 | **0.43**** |
| Pe | **0.25*** | 0.35 | 0.20 | -0.13 | 0.17 | **-0.39*** | **0.55*** | -0.07 | **0.37*** |
| Evap | **0.28*** | 0.29 | 0.27 | -0.13 | 0.05 | **-0.36*** | **0.59**** | -0.05 | **0.43*** |
| Rh | -0.23 | -0.27 | -0.32 | 0.26 | -0.13 | 0.21 | -0.33 | 0.14 | -0.10 |
| Cbl | -0.24 | -0.12 | 0.45 | -0.20 | 0.03 | -0.16 | 0.17 | -0.32 | -0.13 |
| Dublin 2019 - Total Pollen | | | | | | | | | |
|  | January | February | March | April | May | June | July | August | September |
| T_max_ | -0.18 | **0.89**** | **0.15*** | **0.41**** | **0.62**** | **0.56**** | -0.12 | 0.34 | 0.22 |
| T_min_ | -0.26 | 0.29 | -0.12 | 0.25 | 0.30 | 0.35 | -0.29 | -0.15 | -0.24 |
| T_mean_ | **-0.19**** | **0.66*** | 0.08 | **0.45*** | **0.54*** | **0.53**** | -0.32 | 0.02 | -0.19 |
| T_mean_10_ | -0.60 | **0.64**** | -0.07 | -0.06 | 0.05 | -0.14 | **-0.79**** | -0.07 | **0.44**** |
| Gmin | -0.17 | 0.32 | -0.26 | -0.06 | 0.18 | 0.16 | -0.15 | **-0.34*** | -0.08 |
| Rain | **0.34*** | -0.43 | 0.19 | -0.32 | **-0.36*** | -0.34 | -0.36 | **-0.71*** | **-0.53*** |
| Rain_10 | 0.45 | 0.10 | **-0.50*** | 0.05 | **-0.59**** | -0.52 | -0.32 | -0.15 | **0.73**** |
| Wind_S | **0.36*** | -0.13 | 0.03 | -0.11 | -0.21 | -0.16 | **-0.38*** | -0.28 | 0.22 |
| Wind_D | 0.30 | **-0.73**** | -0.28 | 0.19 | -0.04 | -0.12 | 0.21 | -0.18 | **0.40*** |
| Sun | 0.09 | 0.01 | **0.13*** | **0.28*** | 0.11 | **0.42*** | 0.14 | **0.33*** | 0.34 |
| G_rad | 0.26 | 0.24 | -0.07 | **0.32*** | 0.26 | **0.50*** | 0.18 | 0.28 | **0.49*** |
| Soil | -0.17 | **0.74**** | **0.04*** | 0.37 | **0.71**** | **0.47**** | -0.03 | **0.37*** | 0.19 |
| Pe | 0.08 | **0.56**** | **-0.03*** | **0.50**** | **0.39*** | **0.46**** | 0.10 | 0.26 | **0.64**** |
| Evap | 0.12 | **0.62**** | -0.06 | **0.48**** | **0.34*** | **0.44*** | 0.11 | 0.24 | **0.67**** |
| Rh | 0.13 | -0.39 | 0.11 | **-0.42*** | -0.22 | **-0.4*** | -0.18 | -0.38 | **-0.64**** |
| Cbl | -0.50 | **0.63**** | -0.19 | **0.43*** | **0.35**** | 0.40 | **0.75**** | **0.35*** | **0.44*** |

**Table S5** Spearman´s rank correlation coefficients between monthly total pollen data and meteorological parameters for Dublin 2018-2019

**significance at the 95% level, **significance at the 99% level*
