# Supplementary material for: A comprehensive aerobiological study of the airborne pollen in the Irish environment
Source: Aerobiologia (Bologna). 2022 Jul 28;38(3):343–66. doi: 10.1007/s10453-022-09751-w (PMC9526691; doi:10.1007/s10453-022-09751-w)
Supplement: Supplementary file 8 — Supplementary file8 (DOCX 20 KB) [file 10453_2022_9751_MOESM8_ESM.docx]

| Carlow 2018 - Total Pollen | | | | | | | | | |
| --- | --- | --- | --- | --- | --- | --- | --- | --- | --- |
|  | January | February | March | April | May | June | July | August | September |
| T_max_ | **-** | **-** | **-** | -0.46 | **0.64**** | -0.07 | **0.61**** | 0.01 | **0.47*** |
| T_min_ | **-** | **-** | **-** | -0.56 | **0.62**** | -0.22 | -0.11 | 0.11 | 0.27 |
| T_mean_ | **-** | **-** | **-** | **-0.64*** | **0.66**** | -0.13 | **0.58*** | 0.07 | 0.37 |
| T_mean_10_ | **-** | **-** | **-** | 0.06 | **0.19**** | 0.16 | **0.66*** | **-0.55*** | 0.16 |
| Gmin | **-** | **-** | **-** | -0.44 | **0.55*** | -0.35 | -0.23 | -0.03 | 0.31 |
| Rain | **-** | **-** | **-** | -0.28 | -0.20 | 0.02 | -0.42 | -0.10 | -0.22 |
| Rain_10 | **-** | **-** | **-** | -0.46 | -0.42 | **-0.32*** | **-0.70*** | -0.06 | -0.02 |
| Wind_S | **-** | **-** | **-** | -0.48 | -0.23 | 0.14 | 0.02 | -0.07 | 0.05 |
| Wind_D | **-** | **-** | **-** | -0.24 | **-0.24*** | -0.08 | -0.11 | -0.15 | 0.09 |
| G_rad | **-** | **-** | **-** | -0.01 | 0.27 | 0.12 | **0.56**** | -0.14 | **0.43*** |
| Soil | **-** | **-** | **-** | **-0.51*** | **0.65**** | -0.02 | **0.75**** | -0.09 | **0.50**** |
| Pe | **-** | **-** | **-** | -0.38 | **0.40*** | 0.12 | **0.61**** | -0.18 | **0.52**** |
| Evap | **-** | **-** | **-** | -0.40 | 0.31 | 0.12 | **0.60**** | -0.18 | **0.52**** |
| Rh | **-** | **-** | **-** | -0.35 | 0.07 | -0.11 | **-0.41**** | 0.09 | -0.32 |
| Cbl | **-** | **-** | **-** | -0.15 | 0.02 | -0.09 | 0.55 | 0.05 | 0.18 |
| Carlow 2019 - Total Pollen | | | | | | | | | |
|  | January | February | March | April | May | June | July | August | September |
| T_max_ |  | **0.27**** | 0.16 | **0.58**** | **0.51*** | 0.14 | -0.41 | 0.11 | 0.10 |
| T_min_ |  | -0.06 | -0.23 | 0.26 | **0.56**** | 0.05 | **-0.53**** | 0.21 | -0.04 |
| T_mean_ |  | **0.13*** | -0.02 | **0.55**** | **0.61**** | 0.13 | **-0.69**** | 0.18 | 0.00 |
| T_mean_10_ |  | **0.38**** | -0.25 | -0.01 | **0.40*** | -0.14 | -0.25 | **0.71**** | 0.34 |
| Gmin |  | 0.08 | -0.21 | -0.03 | **0.52**** | -0.04 | **-0.40*** | 0.17 | -0.14 |
| Rain |  | -0.09 | 0.11 | -0.45 | -0.11 | -0.40 | -0.17 | 0.01 | -0.31 |
| Rain_10 |  | **-0.33*** | -0.08 | 0.25 | -0.18 | **-0.29*** | -0.22 | **-0.61**** | -0.09 |
| Wind_S |  | -0.36 | 0.23 | -0.22 | 0.30 | -0.14 | -0.43 | 0.00 | 0.17 |
| Wind_D |  | -0.23 | **-0.22*** | 0.01 | 0.28 | -0.02 | 0.32 | -0.28 | 0.28 |
| G_rad |  | **0.35*** | 0.20 | **0.42**** | -0.13 | 0.01 | **0.22**** | 0.09 | 0.36 |
| Soil |  | **0.19*** | -0.06 | **0.53**** | 0.46 | 0.23 | -0.01 | **0.51**** | 0.11 |
| Pe |  | **0.20**** | 0.18 | **0.52**** | 0.12 | 0.05 | 0.13 | 0.13 | 0.48 |
| Evap |  | **0.24**** | 0.28 | **0.48**** | 0.10 | 0.01 | 0.13 | 0.13 | **0.49*** |
| Rh |  | -0.08 | -0.05 | **-0.37*** | 0.01 | -0.29 | **-0.28*** | -0.17 | **-0.56*** |
| Cbl |  | **0.25*** | -0.26 | 0.16 | -0.11 | 0.26 | **0.54**** | -0.25 | 0.32 |

**Table S6** Spearman´s rank correlation coefficients between monthly total pollen data and meteorological parameters for Carlow 2018-2019

**significance at the 95% level, **significance at the 99% level*
