# Supplementary material for: A comprehensive aerobiological study of the airborne pollen in the Irish environment
Source: Aerobiologia (Bologna). 2022 Jul 28;38(3):343–66. doi: 10.1007/s10453-022-09751-w (PMC9526691; doi:10.1007/s10453-022-09751-w)
Supplement: Supplementary file 9 — Supplementary file9 (DOCX 18 KB) [file 10453_2022_9751_MOESM9_ESM.docx]

| Dublin 2018 | | | | Dublin 2019 | | | |
| --- | --- | --- | --- | --- | --- | --- | --- |
|  | Total Pollen  (grains/m3) | Prevalent Pollen Types | %  Contribution |  | Total Pollen  (grains/m3) | Prevalent Pollen Types | % Contribution |
| **January** | 55 | *Corylus* | 39% | **January** | 151 | *Alnus* | 62% |
|  |  | Cupressaceae/Taxaceae | 22% |  |  | *Corylus* | 21% |
|  |  | *Alnus* | 17% |  |  | Cupressaceae/Taxaceae | 14% |
| **February** | 587 | Cupressaceae/Taxaceae | 60% | **February** | 3411 | Cupressaceae/Taxaceae | 53% |
|  |  | *Alnus* | 22% |  |  | *Alnus* | 38% |
|  |  | *Corylus* | 10% |  |  | *Corylus* | 3% |
| **March** | 602 | Cupressaceae/Taxaceae | 43% | **March** | 1775 | Cupressaceae/Taxaceae | 47% |
|  |  | *Fraxinus* | 33% |  |  | *Betula* | 32% |
|  |  | *Alnus* | 15% |  |  | *Alnus* | 5% |
| **April** | 5317 | *Betula* | 48% |  |  | *Ulmus* | 4% |
|  |  | *Fraxinus* | 25% |  |  | *Salix* | 4% |
|  |  | Cupressaceae/Taxaceae | 19% | **April** | 6474 | *Betula* | 61% |
| **May** | 1323 | Cupressaceae/Taxaceae | 35% |  |  | Cupressaceae/Taxaceae | 16% |
|  |  | Poaceae | 17% |  |  | *Platanus* | 7% |
|  |  | *Pinus* | 15% |  |  | *Quercus* | 7% |
|  |  | *Quercus* | 11% | **May** | 4949 | *Pinus* | 28% |
|  |  | Urticaceae | 7% |  |  | Cupressaceae/Taxaceae | 20% |
|  |  | *Betula* | 5% |  |  | *Quercus* | 19% |
| **June** | 6394 | Poaceae | 47% |  |  | Poaceae | 13% |
|  |  | Urticaceae | 36% |  |  | *Betula* | 7% |
|  |  | Cupressaceae/Taxaceae | 12% |  |  | Urticaceae | 4% |
| **July** | 637 | Urticaceae | 50% | **June** | 14966 | Poaceae | 52% |
|  |  | Poaceae | 34% |  |  | Urticaceae | 34% |
|  |  | Cupressaceae/Taxaceae | 5% |  |  | *Quercus* | 8% |
| **August** | 760 | Urticaceae | 81% | **July** | 14102 | Poaceae | 56% |
|  |  | Cupressaceae/Taxaceae | 8% |  |  | Urticaceae | 36% |
|  |  | Poaceae | 7% | **August** | 4337 | Urticaceae | 72% |
| **September** | 361 | Urticaceae | 58% |  |  | Poaceae | 17% |
|  |  | Poaceae | 11% | **September** | 1907 | Urticaceae | 69% |
|  |  | Cupressaceae/Taxaceae | 10% |  |  | Poaceae | 13% |
|  |  | *Mercurialis* | 9% |  |  | Cupressaceae/Taxaceae | 2% |

**Table S7** Monthly distribution of pollen concentrations for Dublin 2018-2019
