# Supplementary material for: A comprehensive aerobiological study of the airborne pollen in the Irish environment
Source: Aerobiologia (Bologna). 2022 Jul 28;38(3):343–66. doi: 10.1007/s10453-022-09751-w (PMC9526691; doi:10.1007/s10453-022-09751-w)
Supplement: Supplementary file 10 — Supplementary file10 (DOCX 17 KB) [file 10453_2022_9751_MOESM10_ESM.docx]

| Carlow 2018 | | | | Carlow 2019 | | | |
| --- | --- | --- | --- | --- | --- | --- | --- |
| Month | Total Pollen (grains/m3) | Prevalent Pollen Types | % Contribution |  | Total Pollen (grains/m3) | Prevalent Pollen Types | % Contribution |
| **February** | - | - | - | **February** | 1463 | *Alnus* | 71% |
|  |  |  |  |  |  | *Corylus* | 14% |
|  |  |  |  |  |  | Cupressaceae/Taxaceae | 13% |
| **March** | - | - | - | **March** | 1297 | Cupressaceae/Taxaceae | 38% |
|  |  |  |  |  |  | *Alnus* | 26% |
|  |  |  |  |  |  | *Salix* | 13% |
| **April** | 1537 | *Fraxinus* | 78% |  |  | *Betula* | 12% |
|  |  | *Betula* | 16% | **April** | 8609 | *Betula* | 78% |
|  |  | Cupressaceae/Taxaceae | 5% |  |  | *Quercus* | 9% |
| **May** | 3165 | Poaceae | 32% |  |  | Cupressaceae/Taxaceae | 4% |
|  |  | *Pinus* | 19% | **May** | 5109 | Poaceae | 29% |
|  |  | *Betula* | 16% |  |  | *Pinus* | 19% |
|  |  | *Fraxinus* | 11% |  |  | *Quercus* | 18% |
|  |  | *Quercus* | 9% |  |  | *Betula* | 7% |
| **June** | 55832 | Poaceae | 86% |  |  | Urticaceae | 6% |
|  |  | Urticaceae | 11% |  |  | *Plantago* | 6% |
| **July** | 4497 | Poaceae | 77% | **June** | 61398 | Poaceae | 81% |
|  |  | Urticaceae | 16% |  |  | Urticaceae | 13% |
| **August** | 850 | Poaceae | 36% | **July** | 3929 | Poaceae | 68% |
|  |  | Urticaceae | 56% |  |  | Urticaceae | 25% |
| **September** | 339 | Urticaceae | 58% | **August** | 1650 | Poaceae | 47% |
|  |  | Poaceae | 28% |  |  | Urticaceae | 46% |
|  |  | *Plantago* | 6% | **September** | 823 | Poaceae | 60% |
|  |  | Cupressaceae/Taxaceae | 5% |  |  | Urticaceae | 32% |

**Table S8** Monthly distribution of pollen concentrations for Carlow 2018-2019
